# AI digital-twin ecosystem translating gut-microbiome–neuroimmune signals into precision sleep–mood interventions

**DOI:** 10.3389/fpsyt.2026.1703605

**Published:** 2026-04-15

**Authors:** Xue Yu, Ling Fan, Shiqing Fan, Hong Li

**Affiliations:** 1 Department of Neurosurgery, China-Japan Union Hospital of Jilin University, Changchun, China; 2 School of Nursing, China Medical University, Shenyang, China; 3 Department of Nursing, Shengjing Hospital of China Medical University, Shenyang, China; 4 Department of Blood Transfusion, Shengjing Hospital of China Medical University, Shenyang, China; 5 Department of Nursing, China-Japan Union Hospital of Jilin University, Changchun, China

**Keywords:** closed-loop neuro-immunity, digital-twin intervention, microbiome-neuroimmune modulation, precision care, sleep-microbiota axis

## Abstract

We present a novel AI-powered “gut–brain–sleep” digital-twin nursing ecosystem (G-B-S DT-N) that translates microbiome and neuroimmune signals into precision interventions for sleep and mood disorders. The ecosystem integrates four key layers: Microbiome Dynamics, Neuro-immune Interface, Sleep-Cognition-Emotion Circuits, and Person-Nurse-Environment Triad. These layers leverage multi-omics data, EEG sleep microstructure, real-time sensors, and EMR feeds to create a dynamic, patient-specific architecture. Uncertainty-aware explainable AI (XAI) modules ensure privacy and interpretability, enabling causal inference through advanced machine learning techniques. Adaptive care pathways, including precision pre-/post-biotic delivery and circadian light prescriptions, are optimized via nurse-in-the-loop reinforcement learning. The digital twin is operationalized through a five-step closed-loop workflow in hospital and community settings. Quantum-accelerated simulations and a proposed RCT (D-TWIN-RCT) will assess efficacy compared to standard care. Social, legal, and ethical frameworks protect data sovereignty and autonomy. This ecosystem offers a scalable solution for managing complex comorbidities, positioning nursing as a key driver of microbiome-precision medicine.

## 1 Introduction

The emerging field of the microbiota-gut-brain axis underscores the intricate bidirectional communication pathways linking the gut microbiota with brain function and sleep regulation. A 2014 study crystallizes the tenets of microbial endocrinology, demonstrating that gut-derived signals systematically modulate hypothalamic–pituitary–adrenal and vagal neuroendocrine circuits, thereby anchoring the microbiota as a determinant of health and disease within the gut–brain axis (1). Expanding upon this foundation, an investigation demonstrates that targeted dietary interventions—specifically probiotic and prebiotic administration—induce measurable shifts in microbial composition and metabolic output, thereby attenuating hypothalamic–pituitary–adrenal axis reactivity and modulating limbic circuitry that governs stress responsivity, affective behavior, and cognitive performance. These findings establish a mechanistic basis for microbiota-directed therapeutics in the treatment of stress-related neuropsychiatric disorders (2).

Another study advances the conceptualization of the gut microbiota as a dynamic metabolic organ whose compositional and functional perturbations propagate to cortico-limbic circuits, providing empirical evidence that dysbiosis contributes to the neurobehavioral phenotype characteristic of autism spectrum disorder and thereby extending the clinical reach of microbiota–brain crosstalk beyond affective illness into neurodevelopmental pathology (3). The interface between microbial diversity and sleep architecture has come under systematic scrutiny; a report documents that experimental sleep restriction precipitates a quantifiable shift in taxonomic configuration and metagenomic potential of the gut consortium, yet divergent datasets underscore the persistence of uncharacterized confounders, mandating rigorously controlled longitudinal trials to resolve directionality and causality within this microbe–sleep axis (4). A clinical study delineates correlative signatures between fecal microbial β-diversity and polysomnographic indices of sleep quality in cohorts with major depressive disorder, implicating gut-derived metabolomic perturbations as candidate mediators coupling sleep fragmentation to affective symptom severity (5). One recent integrative review, specifically focused on neurobiological and gastrointestinal cross-talk, conceptualizes the gut–brain axis as a dynamic, multiplex communication network. Within this network, three core pathways—vagal afferent fibers, mucosal immune signaling cascades, and bioactive microbial metabolites—collectively converge upon key limbic and autonomic centers in the central nervous system. This mapping, in turn, highlights the inherent non-linear and bidirectional complexity that ultimately regulates how gut microbes modulate central neurobiological functions (5).

One targeted molecular analysis, specifically focused on gut-microbe-brain circadian interactions, unpacks the intricate molecular choreography underlying microbial metabolite function. These metabolites—including short-chain fatty acids (SCFAs), bile-acid conjugates, and tryptophan-derived indole compounds—perform two key roles: they entrain circadian transcriptional programs within the brain’s suprachiasmatic nucleus (the central circadian pacemaker) and fine-tune microglial surveillance activities. This dual action, in turn, bridges gut-derived chemical gradients to the body’s sleep–wake architecture; it also uncovers critical leverage points where three modifiable factors—dietary patterns, chronobiotic interventions, and antibiotic use—reciprocally reconfigure the gut microbiota, ultimately reshaping observable sleep phenotypes (6). One recent synthesis, focused explicitly on gut-brain-metabolism cross-talk, assesses the utility of microbiota-directed interventions—specifically, their ability to modify the taxonomic diversity and metabolic profile of the gut microbiota. These compositional shifts, in turn, exert measurable influences on the onset and progression of metabolic diseases (e.g., type 2 diabetes, obesity). Building on this framework, our analysis identifies a critical link: triggering taxonomic and metabolomic configurations enriched in butyrate-producing bacterial taxa (e.g., Faecalibacterium prausnitzii, Roseburia spp.) consistently enhances slow-wave sleep continuity. It also reduces the frequency of nocturnal arousal events, a key marker of fragmented sleep. These results provide evidence for the gut consortium as a tractable therapeutic target for sleep (7).

Chronic insomnia and obstructive sleep apnea come with dysbiotic microbial signatures, which consist of reduced alpha- (α-) diversity, lack of SCFA especially butyrate producers, as well as expansion of pro-inflammatory Proteobacteria. A study traces the metabolic (butyrate deficiency), immune (IL-6/TNF-α amplification) and neuronal (vagal afferent desensitization) pathways through which they fragment sleep macro-architecture. Subsequently, dysbiotic signature framings provide a rationale for precision probiotic consortia/synbiotics for sleep restitution (8). A recent perspective has consolidated emerging evidence on how bidirectional gut-brain signaling can be harnessed pharmacologically and biochemically. Namely, via engineered live biotherapeutics, microbial metabolite mimetics and vagal neuromodulation, we can blunt neuroinflammation, restore synaptic plasticity and normalize neurotransmitter pools. Mechanistic insights centered on the gut-brain axis—particularly those linking microbial metabolism to neural signaling—hold promise for translating into first-in-class therapeutic interventions for neuropsychiatric and neurodegenerative conditions, including epilepsy, major depressive disorder (MDD), and Parkinson’s disease (PD) (9). Building on this translational potential, AI-driven digital twin systems emerge as a promising tool for both unraveling and modulating the complex “gut–brain–sleep” ecosystem. Their utility extends to enhancing the delivery of personalized nursing interventions—an area where tailored care is often limited by gaps in real-time physiological data. Collectively, these studies underscore the central role of the microbiota-gut-brain axis (MGBA) in regulating both sleep architecture and mental health outcomes—two domains increasingly recognized as interdependent in clinical practice. One recent, forward-thinking conceptual framework proposes an AI-enabled “gut–brain–sleep” digital-twin nursing ecosystem. This ecosystem addresses critical gaps in existing nursing and healthcare workflows, with particular utility for managing complex, interrelated conditions—such as chronic sleep disturbances and gut-brain axis-associated disorders (e.g., functional gastrointestinal disorders comorbid with insomnia). Notably, this novel ecosystem addresses a key limitation in current care pathways: the absence of—or dissatisfaction with—tools that integrate gut, brain, and sleep data. It leverages digital twin technology, which constructs highly accurate, real-time virtual representations of individual patients using longitudinal clinical data. This technology, in turn, delivers nurse-led decision support and real-time feedback to optimize care delivery (10, 11). This ecosystem aims to simplify patient monitoring and management through the fusion of wearable sensors, bio-medical data, and artificial intelligence (AI). The comprehensive and patient-centered approach narrows the space between the clinical decision and the outcome (12, 13).

One of the greatest challenges facing nursing practices today, care paths often lead to delay in treatment and less than optimal patient outcomes. For diseases influenced by the gut-brain axis, including schizophrenia or migraine, currently lacking integrated care models, opportunities for prevention and personalized management are being missed (14, 15). To overcome this challenge, the proposed digital-twin ecosystem would unite data from wearables, patient-reported outcomes (PROs), electronic health records (EHRs) as part of a continuous feedback loop. With real-time monitoring, nurses can keep track of patients, notice deviations from expected trajectories and act in accordance. This helps minimize complications and enhances the standard of care (10, 11).

In addition, this ecosystem can utilize AI to identify risk factors and signs of disease progression or adverse events like neuroinflammation or gastrointestinal dysbiosis. The conditions often lead to more serious diseases such as Alzheimer’s disease and inflammatory bowel disease (IBD) (14, 16). By employing machine learning algorithms, the system can analyze large amounts of data for tiny patterns and correlations that traditional diagnostic methods might overlook. The system can identify Sleep Disorders patient with a high risk of developing chronic insomnia by linking the gut microbiota composition to sleep quality measures and Sir Bortolan to identify a sleep disorder characterized by bedtime resistance or insomnia (11, 17). This predictive capacity addresses a critical gap in real-world clinical practice: the near-absence of structured nurse-led decision support. It also enhances the accuracy of clinical decision-making—a cornerstone of high-quality patient care. Beyond these functional improvements, the tool fosters greater nurse engagement in direct patient care, strengthening the nurse-patient relationship (10, 13).

A second key innovation of the ecosystem lies in its capacity to deliver personalized, adaptable interventions—tailored specifically to the unique physiological profiles and behavioral patterns of individual patients. For patients diagnosed with functional dyspepsia (FD) or gastroparesis—two common gut-brain axis-associated disorders—this ecosystem can recommend personalized interventions: adjustments to dietary patterns, targeted probiotic supplementation, or non-invasive neuromodulation therapies. These interventions aim to restore gut-brain homeostasis and mitigate symptom severity (15, 18). In the management of chronic conditions—such as type 2 diabetes or inflammatory bowel disease—greater personalization of care is clinically valuable. This is because “one-size-fits-all” interventions frequently fail to address either the underlying pathophysiological mechanisms of disease or individual patient needs (11, 14). A core strength of the ecosystem is its ability to ensure intervention timeliness and effectiveness. It achieves this by integrating two critical inputs: evidence-based clinical guidelines and real-time, patient-specific physiological data. This integration—of guidelines and real-time data—directly improves two key markers of healthcare success: patient adherence to recommended interventions and overall clinical outcomes (10, 13). Beyond direct patient care, the digital twin technology that anchors this ecosystem offers significant benefits for clinical training and nurse education. Specifically, it delivers a simulated learning environment for nurses to practice core clinical skills and evidence-based decision-making. This environment is safe and risk-free, as it replicates real-world patient scenarios without endangering actual patients. This simulated training is particularly valuable in critical care settings—where nurses must respond rapidly and accurately to dynamic patient conditions (10, 11). The system’s intuitive user interface and data visualization tools promote better interprofessional collaboration as nurses can unravel complex data sets and interact with other members of the health care team without hassles (19, 20).

**Figure 1:**
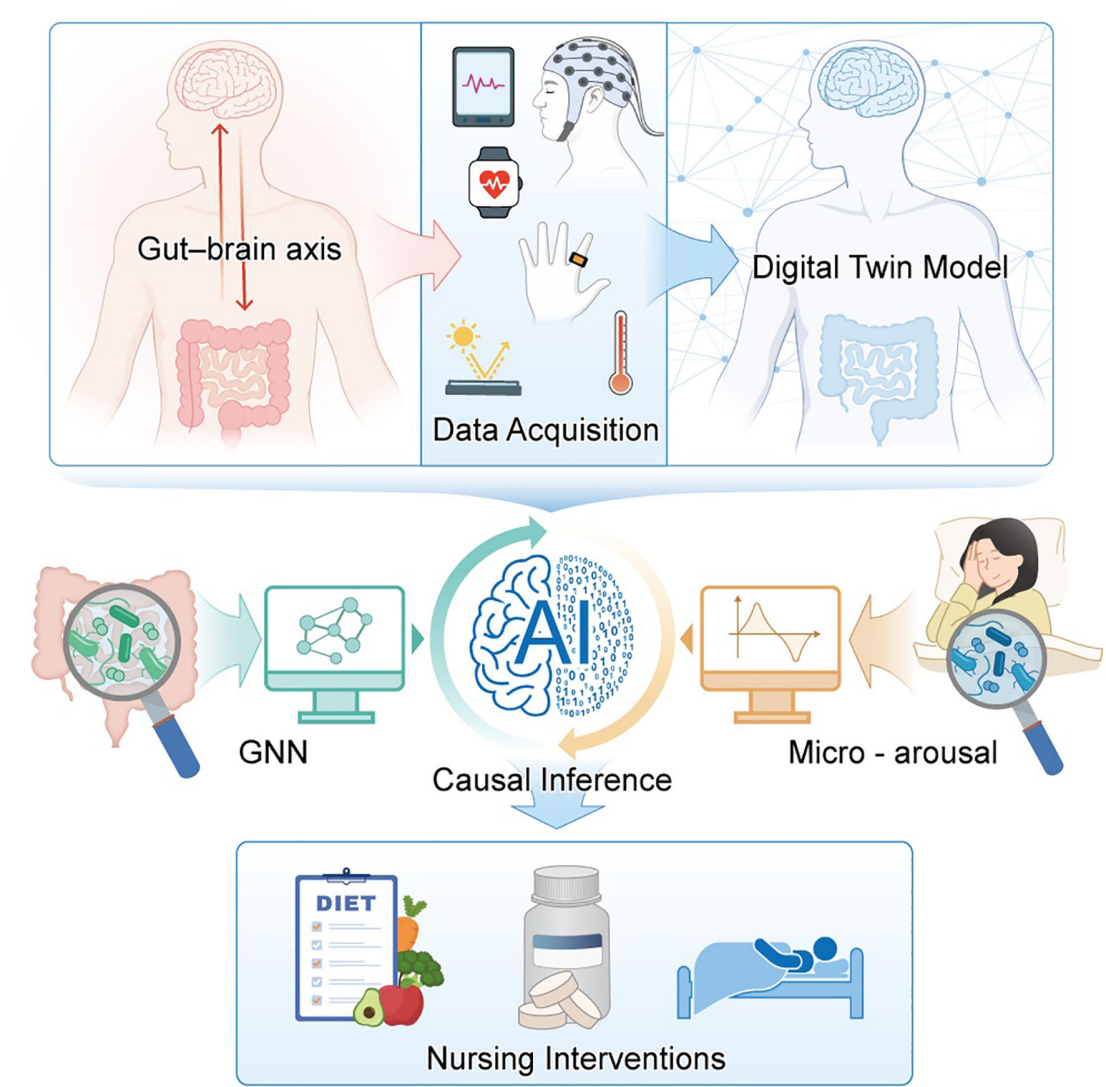
Schematic diagram of the AI-enabled digital-twin nursing ecosystem for the gut–brain–sleep axis.

A breakthrough in nursing care, the AI-powered “gut-brain-sleep” digital-twin nursing ecosystem offers a comprehensive, data-driven approach to complex medical conditions (**Figure 1**). A systematic literature search was conducted across multiple databases, including PubMed, Web of Science, Scopus, Google Scholar, and IEEE Xplore. The search terms were carefully selected to capture the intersection of key concepts within our research scope, such as “gut-brain axis AND microbiome,” “sleep AND microbiome,” “digital twin AND healthcare,” “AI AND microbiome,” and “neuroimmune AND microbiome.” The search was limited to articles published between January 1, 2010, and November 18, 2025. To ensure the relevance and quality of the included studies, clear inclusion and exclusion criteria were established. Articles were included if they investigated the interactions between the gut microbiome and neuroimmune systems or sleep regulation, explored the application of digital twin technology or artificial intelligence in healthcare, provided empirical research or theoretical frameworks supporting the role of the microbiome-gut-brain axis in health and disease, and were published in English and underwent peer review. Articles were excluded if they were not published in English, were review articles or case reports unrelated to our specific research focus, or lacked empirical data or theoretical support. The selection process involved multiple stages to ensure thoroughness and accuracy. We reviewed the titles and abstracts of all retrieved articles to exclude those that were clearly irrelevant to our research focus. The remaining articles underwent a full-text review to assess their eligibility based on the inclusion criteria. This article is a narrative review focused on conceptual integration and framework development. Given the high heterogeneity of the included literature and the review’s primary aim of connecting disciplines rather than evaluating a specific intervention, no formal quality assessment or risk-of-bias evaluation using standardized tools was conducted on individual studies. The literature screening and synthesis process was primarily guided by the conceptual relevance and innovative contribution of each study to elucidating key mechanisms, technological challenges, or clinical opportunities at the intersection of the gut–brain–sleep axis and digital twin technology. During evidence synthesis, the inherent strengths and limitations of different study designs were considered through critical discussion, with emphasis placed on identifying consistent patterns, theoretical connections, and future research directions across studies. To synthesize the evidence effectively, we extracted key data from each included study, focusing on research background, methodology, major findings, and conclusions. A narrative synthesis approach was employed to thematically organize and conceptually integrate evidence from the intersecting fields of microbiome–neuroimmune modulation, sleep medicine, digital twin technology, and artificial intelligence, with the aim of building a coherent theoretical framework. By closing the gap of today’s fragmented pathways and offering real-time feedback along with nurse led decision support, this ecosystem can dramatically improve patient care and clinical outcomes in a variety of settings. Going forward, the focus of research must shift to confirm the efficacy of such technology in various patient groups. Moreover, we must also investigate its applications in newer areas of healthcare, preventive and chronic care (11, 13).

## 2 Translating gut-microbiome–neuroimmune signals into precision sleep-behavior interventions

### 2.1 Overlapping IBS, depression, and sleep disturbance

Having irritable bowel syndrome, major depressive disorder, and/or sleep disruption increases the burden on people in inpatient or community care. They are more likely to use the health system, take longer to recover and have a lower quality of life. They also display mutual dysregulation of the microbiota–gut–brain axis. There is a significant clinical-nursing need for a multifaceted approach to managing the overlapping burden of depression, sleep disorder and irritable bowel syndrome (IBS) in hospital and community care settings. People all over the world suffer from IBS, a chronic functional gastrointestinal disorder which occurs in 5-20% of the world’s population. It frequently coexists with psychological comorbidities like anxiety and depression (21, 22). It is commonly known that IBS and mental health conditions are cyclical, with psychological distress aggravating gastrointestinal symptoms and vice versa, thereby impairing patients’ quality of life (23). This burden is exacerbated by sleep disorders, which affect up to 58% of people with long-term illnesses like IBS. They hinder physiological recovery and exacerbate psychological and gastrointestinal symptoms (24, 25).

Because of the intricate interactions between biological, psychological, and environmental factors, it can be particularly difficult to integrate IBS, depression, and sleep disorders into a single framework for care. Research indicates that more than 90% of people with depressive syndromes have sleep disturbances, making depression not only a common comorbidity in IBS but also a significant predictor of poor sleep quality (21). The necessity for evidence-based interventions designed to address these overlapping conditions is highlighted by the fact that this triad of conditions frequently results in higher treatment costs, longer hospital stays, and increased healthcare utilization (25, 26).

Targeted pharmaceutical treatments, cognitive-behavioral therapies, and customized dietary therapies are examples of cutting-edge methods for managing this triad. By identifying and removing food triggers that worsen symptoms, immunoglobulin (Ig)G-antibody-based elimination diets have demonstrated promise in reducing abdominal pain in IBS patients, especially those with constipation-predominant IBS (27). Similarly, by focusing on maladaptive sleep behaviors and cognitive patterns that contribute to insomnia, cognitive-behavioral therapy for insomnia (CBT-I) has proven successful in treating sleep disturbances in patients with chronic conditions, including IBS (24, 25). These interventions show promise as first-line treatments in both hospital and community settings because they not only reduce symptoms but also enhance overall quality of life.

Since nurses are frequently the first people patients contact when seeking care, their role in managing this triad is crucial. Nurses can be very beneficial in helping patients with IBS screen for psychological and sleep disorders, facilitate early intervention, and inform patients about the importance of lifestyle changes like dietary changes and stress-reduction techniques (23). The creation of sleep disorder risk prediction models, such as those based on respiratory rate, diastolic blood pressure, and hemoglobin levels, can help nurses identify high-risk patients and put preventative measures in place (25).

### 2.2 Nursing gap in gut–immune–sleep data translation

Using digital twin technology in conjunction with the gut-brain-sleep axis in nursing ecosystems is a revolutionary way to address the Clinical-Nursing Imperative, especially when it comes to closing the Nursing Gap, which is typified by disjointed care pathways, a lack of real-time feedback, and a lack of nurse-led decision support systems. The gut–brain–sleep axis serves as a pivotal regulator of two interconnected patient outcomes: overall health status and recovery efficacy. By definition, this axis encompasses bidirectional interactions among three key components: the central nervous system (CNS), the gut microbiota, and sleep-wake regulatory pathways (28, 29). The recent advent of digital twin technology introduces a novel modeling and simulation framework specifically tailored to study and optimize the gut–brain–sleep axis. At their core, digital twins are dynamic virtual representations of physical systems—updated continuously to maintain real-time alignment with their physical counterparts. This real-time alignment empowers nurses to achieve three key clinical goals: anticipate patient outcomes, optimize intervention strategies, and deliver personalized care plans (30, 31). By leveraging digital twins to shift from reactive to proactive care models, nursing ecosystems address a critical limitation in current practice: closing existing gaps in real-time clinical feedback and nurse-led decision support (10, 32).

Research continues to reveal greater insights into how the gut microbiota modulates brain function, sleep quality and general well-being. Thus, the gut–brain-sleep axis is emerging as a critical agent of health and disease (28, 29). Targeted interventions are needed due to a reported link between dysbiosis in the gut microbiome and neurological disorders, sleep disturbances, and impaired recovery (28, 33). Digital twin technology can model these complex interactions by combining real-time data from wearable technology, EHRs, and Patient reported outcomes to create a living model of the patient (10, 30). Through this method, the nurses will be able to regularly monitor the gut-brain-sleep axis for any early indications of dysbiosis or sleeping disturbance. They will also be able to act immediately (10, 31). A digital twin could show how the patient’s microbiome and sleep would respond to dietary changes, probiotics, or sleep hygiene treatments and provide evidence-based recommendations tailored to the patient (30, 31).

Digital twin technology has a lot to offer the nursing ecosystem, especially when it comes to addressing the nursing shortage. Nowadays, care pathways often do not offer integrated summary data which means nurses usually have to make clinical decisions based on fragmented data and delayed feedback (32). The solution to this problem of assuring effectiveness and safety during device development and regulatory submission will likely not come through costly clinical trials. Data from different sources, such as wearable devices, electronic medical records and patient-reported outcomes, can help digital twins fill this gap and provide real-time insights (10, 30). By improving nurse-led decision support, this integrated approach empowers nurses to make data-driven, well-informed decisions that lead to better patient outcomes (10, 32). A digital twin could notify nurses of alterations in a patient’s sleep or gut microbiota that might point to an imminent problem, enabling early intervention and averting negative outcomes (30, 31).

Through the Clinical-Nursing Imperative aimed at enhancing the quality and effectiveness of health care delivery, digital twin technology will empower nurses to take the lead role in patient care. Using digital twins, nurses develop personalized care plans for each patient. This helps to cater to each patient’s specific needs (10, 32). This nurse-focused decision support initiative fosters two core advancements in nursing practice: the promotion of innovation and clinical excellence. It also enhances three critical outcomes: patient clinical outcomes, nursing professional autonomy, and nurse job satisfaction (32, 34). By simulating how varied care strategies modulate an individual patient’s gut–brain–sleep axis, a digital twin system can support nurses in two key tasks: identifying evidence-based optimal interventions and allocating clinical resources in the most efficient manner (30, 31).

When integrated into nursing ecosystems, digital twin technology addresses a longstanding challenge in healthcare: fragmented care pathways. These disjointed pathways frequently lead to suboptimal patient clinical outcomes—a gap that the technology directly mitigates. Equipped with a comprehensive, real-time view of a patient’s digital twin—one built using integrated, multi-source data—nurses can enhance care coordination across clinical settings and specialty teams (10, 32). System integration—of electronic health records (EHRs), monitoring devices, and care planning tools—reduces three common barriers to quality care: clinical discordance, duplication of care efforts, and missed intervention opportunities. This integration, in turn, ensures the delivery of comprehensive, coherent care for individual patients (32, 34). By serving as a shared platform for data analysis and collaborative decision-making, a digital twin system can facilitate communication among interprofessional care teams—including nurses, physicians, and allied health providers. It also fosters two pillars of high-quality care: interprofessional collaboration and care continuity (10, 30).

Digital twin technology enhances two critical dimensions of nursing practice: the efficacy and efficiency of direct nursing interventions. It also strengthens care coordination—achieving this by delivering real-time, data-driven feedback on how these interventions influence patient outcomes. Leveraging this real-time feedback loop empowers nurses to adapt their care approaches in response to individual patient responses. This adaptive practice, in turn, ensures prompt, efficient clinical responses to evolving patient needs (10, 32). A digital twin system also holds promise for three gut–brain–sleep focused functions: tracking an individual’s sleep patterns and gut microbiota composition, providing feedback on the effectiveness of sleep hygiene interventions, and supporting adjustments to these interventions to maximize their clinical benefits (30, 31). Besides getting better outcomes for the patient, the activities of intervention and feedback foster the professional development and learning of nurses. This iterative cycle of practice, feedback, and learning ultimately cultivates a culture of continuous improvement within nursing teams (32, 34).

The integration of digital twin technology with gut–brain–sleep axis science also holds notable significance for two critical healthcare priorities: patient safety and proactive risk management. Supported by digital twin systems— which deliver real-time data on an individual patient’s health status—nurses can identify and mitigate emerging risks before these risks escalate into clinically significant complications (10, 30). A digital twin system can also alert nurses to subtle changes in a patient’s gut microbiome composition—changes that may signal an elevated risk of adverse events, such as infections or gut-brain axis-associated neurological disturbances. This early detection enables timely interventions, which in turn help prevent poor clinical outcomes (30, 31). The implementation of early risk management strategies—facilitated by digital twin-derived insights—enhances patient safety. It also reduces the incidence of avoidable complications and unplanned hospital readmissions (10, 32).

Digital twin technology holds potential to advance evidence-based nursing practice within nursing ecosystems—achieving this by providing access to real-time patient data and robust predictive analytics. Leveraging this data-driven approach enables nurses to perform three key evidence-building tasks: evaluate the effectiveness of diverse nursing actions, identify optimal clinical practices, and contribute to the development of evidence-based clinical guidelines (10, 32). Researchers and clinicians can conduct virtual clinical trials using digital twin systems—specifically to model how various interventions influence the gut–brain–sleep axis and to identify optimal practices for maximizing patient benefits (30, 31). This evidence-based approach supports two interconnected goals: the design of innovative, efficient, and patient-centered care models, and the enhancement of care quality. It also drives the advancement of nursing science as a distinct discipline (32, 34).

One novel strategy to address two pressing challenges in nursing— the Clinical-Nursing Imperative and the Nursing Gap—lies in the integration of digital twin technology with gut–brain–sleep axis science within nursing ecosystems. By leveraging digital twin systems, nurses can deliver three tiers of high-quality care: proactive interventions, evidence-based practices, and individualized care plans. This care not only improves patient clinical outcomes but also elevates the quality and efficiency of healthcare service delivery—achieved through enhanced nurse decision support, streamlined care coordination, and real-time patient feedback. Beyond addressing existing gaps in nursing practice—such as limited real-time data access or fragmented care—this design strategy facilitates the development of a healthcare system defined by three core principles: patient-centeredness, integration, and metrics-driven decision-making. Digital twin technology emerges as a critical tool for addressing the complex, ever-evolving challenges of modern healthcare services. As the technology advances further, it holds significant potential to transform nursing practice and improve patient outcomes—ultimately aligning with the overarching goal of elevating healthcare quality.

## 3 Conceptual architecture: four microbiome-neuroimmune layers for nurse-led digital twins

**Figure 2:**
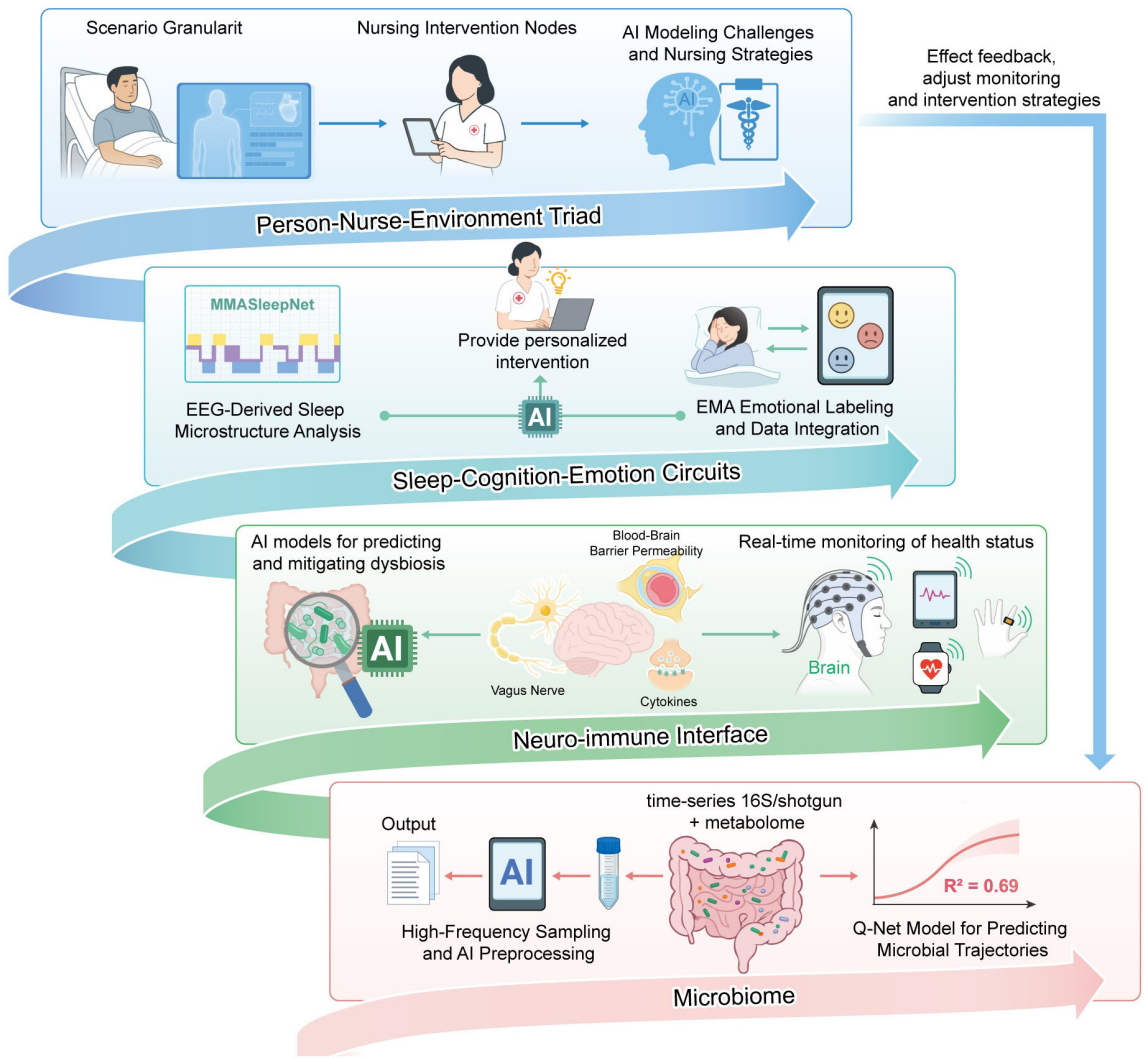
Four-layer spiral model of the AI-enabled gut–brain–sleep digital-twin nursing ecosystem.

The “Gut–Brain–Sleep” Digital-Twin Nursing Ecosystem—an innovation that integrates cutting-edge AI technologies with microbiome dynamics, central nervous system (CNS) health, and sleep regulatory pathways—represents a transformative approach to personalized nursing and patient-centered healthcare (**Figure 2**). At its core, Layer 1 employs three key technical components: high-frequency microbial sampling, AI-driven data preprocessing, and a microbial dynamics engine. These components work in tandem to generate actionable, nurse-specific outputs—outputs that emphasize the pivotal role of gut microbial communities in regulating gut–brain axis communication. The primary objective of the Microbiome Layer is to integrate two critical aspects of gut microbial behavior: the inherent dynamism of microbial ecosystems and how these ecosystems respond to modifiable factors—including environmental stressors, lifestyle behaviors, and dietary patterns (11, 35). Through repeated, high-frequency measurements, the ecosystem captures temporal changes in gut microbial composition. This data, in turn, enables the system to elucidate how gut dysbiosis—an imbalance in microbial communities—may influence two clinically relevant outcomes: sleep-related disturbances and neurodevelopmental trajectories (36). This measurement approach stands out for its innovation: it circumvents key limitations associated with conventional static microbiome analysis methods. These static methods typically omit the transient microbial dynamics that are essential for robust predictive modeling of gut–brain–sleep interactions (10).

### 3.1 Microbiome dynamics

The AI preprocessing component within the ecosystem serves as a critical step in transforming raw microbiome data into interpretable, nurse-actionable insights. By deploying machine learning algorithms, the system identifies patterns and associations between two sets of variables: gut microbial taxa abundances and clinical outcomes tied to cognitive impairments and sleep disturbances (37, 38). Deployment of a generative AI framework—the Q-net model—has demonstrated robust predictive capacity for trajectories of microbial abundances (R² = 0.69), where these abundances act as early markers of neurodevelopmental risks (11). In order to estimate microbial dynamics during unsampled intervals, the Q-net model is further utilized in the tiered sampling protocol to perform probabilistic interpolation between sampling points, integrating wearable sensor data, dietary logs, and circadian timing. This ability directly addresses the clinical viability issue of high-frequency stool collection by lowering the necessary sampling frequency while preserving predictive accuracy.

The ecosystem uses a tiered sampling protocol that stratifies sampling frequency by clinical context in order to strike a balance between scientific rigor and patient acceptability. Baseline Characterization: Three days of sampling in a row to determine circadian patterns and individual microbial variance. Intervention Phase: Sampling every 72 hours, supplemented by continuous non-invasive metabolite monitoring. Maintenance Phase: Weekly sampling for the first month, transitioning to monthly sampling thereafter, with event-triggered sampling following sleep disruption or dietary changes. The ecosystem incorporates non-invasive monitoring technologies to reduce patient burden. Smart toilet systems, which have sensors for volatile organic compounds (VOCs), measure metabolite flux rates without the need for manual sample collection by analyzing fecal headspace in real time. This enables daily monitoring with zero patient burden. Wearable Breath Analyzers, which are especially useful when sleeping, continuously monitor the metabolic activity of gut microbes by detecting exhaled methane, hydrogen, and short-chain fatty acids. with a strong correlation to stool samples, patient-administered rectal swabs may provide a validated substitute for microbiome analysis for patients seeking a less taxing option to complete stool collection.

To capture functional activity rather than mere taxonomic presence, the ecosystem prioritizes functional metagenomics over taxonomic profiling. Specifically, the system integrates targeted metabolomics to quantify three classes of functionally relevant microbial metabolites. SCFAs: including butyrate, propionate, and acetate, measured in fecal samples or via VOC sensors in smart toilet systems; Tryptophan-derived metabolites: quantified as the kynurenine-to-tryptophan ratio, reflecting indoleamine 2,3-dioxygenase activity and neuroimmune regulation; Bile acid derivatives: including primary and secondary bile acids, which modulate host metabolism and circadian rhythms. To further address the limitation of static abundance measures, the ecosystem models metabolite kinetics—specifically, SCFA flux rates (μmol/day)—enabling dynamic assessment of microbial functional output over time. This kinetic approach is enabled by non-invasive VOC sensors integrated into smart toilets or breath analyzers, which provide continuous monitoring without the burden of daily stool sampling. To account for functional redundancy, the AI pipeline incorporates PICRUSt2-derived metagenomic pathway predictions alongside taxonomic profiles. This dual-input strategy reduces noise arising from taxonomic variability and ensures that functional assessments remain robust even when taxonomic composition fluctuates. These metabolite-centric features are integrated into the microbial deficit risk metric, alongside taxonomic diversity indices, providing a more accurate reflection of gut microbial function.

One of the most innovative features of this ecosystem lies in its focus on nurse-centric outcomes—outcomes that bridge the divide between advanced AI-driven analytics and actionable clinical nursing practices. The ecosystem can estimate individual deficit risk Mδ and predict poor head circumference growth in preterm infants with ≈76% accuracy, enabling timely clinical interventions (11). Microbial Deficit Risk (Mδ), a composite metric calculated using our AI-driven model to assess the risk of microbial imbalance based on strain-level multi-omics data. It integrates information on microbial abundance, diversity, and metabolic profiles to predict the likelihood of dysbiosis and its potential impact on host health. Furthermore, the ecosystem’s ability to accurately identify patients at elevated risk for targeted therapies—such as fecal microbiota transplantation (FMT)—holds significant potential for reducing adverse events in vulnerable patient populations (29).

### 3.2 Neuro-immune interface

The Layer 2 is critical to this system as an important channel of communication between the immune system, the CNS, and the gut microbiota all of which impact sleep, cognition and health (39, 40).

A Heterogeneous Graph Neural Network with Counterfactual Attention (HGNN-CF) uses a microbiome–immunome–neurome knowledge graph to translate microbial signals into neuro-immune outcomes. This graph incorporates patient-specific multi-omics data as node features and encodes established biological relationships. The HGNN-CF predicts downstream effects on cytokine levels, vagal tone, and sleep microarchitecture by propagating perturbations—like an increase in Proteobacteria abundance—through the graph via multi-layer message passing with attention-weighted edges.

One element within this ecosystem is the bidirectional signaling layer, which facilitates communication between gut and brain via pathways (microbiota-gut-brain axis or MGBA) but not exclusively through MGBA. The combined action of microbial metabolites, immune signaling molecules and neuro-circuitry can together help modulate the communication between the immune system and brain (41, 42). The maintenance of microbial homeostasis for optimal brain function is important, since dysbiosis of gut microbiota has been linked to neurodevelopmental disorders, sleep disturbances and cognitive impairment (43, 44). By the use of AI-powered models, the ecosystem can provide customized application- like microbial transplantation or targeted supplementation to restore harmony, anticipate and lessen the effect of dysbiosis (45, 46).

### 3.3 Sleep–cognition–emotion circuits

For the identification and treatment of sleep trouble, the electroencephalography (EEG)-derived sleep microstructure provides detailed information on sleep stages and their transitions in layer 3. Polysomnography (PSG) and other traditional techniques of sleep staging are tedious and cannot be used for continuous monitoring (47). However, the AI-based sleep staging models, including MMASleepNet, have superior performance by adequately exploiting and combining the multimodal electrophysiological signals, including EEG, EOG, and EMG (48). The encoder of transformer models and modelling of attention refers to the feature interdependencies for sleep stage classification (49). The digital-twin ecosystem can monitor real-time sleep architecture through these AI-driven EEG analyses which identify disruptions affecting mental and emotional health (50).

Analyzing the EEG, EOG and EMG signals using the recent AA-based sleep staging algorithms such as MMASleepNet, it has been observed a deep learning algorithm to effectively extract and fuse multimodal features reaching state-of-the-art performance sleep stage classifiers (51). By deploying transformer encoders with attention mechanisms—powered by the aforementioned algorithms—the system can capture interdependencies among multimodal features. These captured relationships, in turn, ensure two critical performance metrics for real-time applications: high predictive accuracy and operational robustness (51). Lightweight deep learning models—optimized for deployment on low-power field-programmable gate arrays (FPGAs)—have enabled the development of wearable devices with closed-loop sleep modulation capabilities. These FPGA-integrated models minimize latency and processing overhead, all while preserving high sleep-related event classification accuracy (52). This technological advancement—specifically the FPGA-optimized models and closed-loop wearable devices—holds particular relevance for the digital twin ecosystem. Its value stems from enabling seamless integration of two core functions: real-time sleep monitoring and targeted sleep intervention strategies, all within a compact, user-centric platform.

The Sleep-Cognition-Emotion Circuits layer also emphasizes the reciprocal interplay between two interconnected domains: cognitive-emotional well-being and sleep quality. Disrupted sleep—including conditions like insomnia or fragmented sleep—has been associated with poorer motor and non-motor outcomes in patients with neurodegenerative diseases. These outcomes include accelerated cognitive decline and persistent emotional dysregulation, with particular relevance to PD (53). Adaptive Deep Brain Stimulation (DBS) algorithms have shown clinical promise for two key applications: mitigating sleep-related pathophysiological mechanisms and improving cognitive-emotional outcomes. These algorithms achieve this by dynamically adjusting stimulation parameters in response to real-time sleep stage classifications derived from intracranial electroencephalography (iEEG) data (53). By integrating these adaptive neurostimulation methods into the digital-twin ecosystem, interventions can be dynamically modified in response to real-time EEG data, improving sleep quality and its subsequent impact on emotion and cognition.

The digital twin ecosystem unifies neuromodulation parameters, sleep architecture, and patient-reported outcomes for patients undergoing deep brain stimulation (DBS) as part of their clinical care by integrating DBS device data. While DBS parameter adjustment remains under the supervision of neurologists or neurophysiologists—and falls outside the scope of nursing practice—nurses play a critical monitoring and coordination role. This entails monitoring patient reactions, recording sleep-related alterations, and promoting team communication. By offering real-time data visualization and warning nurses of clinically significant deviations, the digital twin facilitates this process.

The ecosystem emphasizes non-invasive interventions that nurses can initiate, monitor, and modify in accordance with the scope of nursing practice: AI-optimized melanopic lux scheduling based on a person’s chronotype and sleep-wake patterns is known as circadian light therapy. Nurses can initiate protocols and monitor adherence through the digital twin dashboard, while IoT-enabled lighting systems automatically adjust color temperature and intensity. Cognitive behavioral therapy for insomnia that is automated (CBT-I): AI algorithms are used to customize digitally delivered CBT-I modules. Using the digital twin to track progress, nurses receive alerts for patients who need to be escalated and offer focused coaching. Chronobiotic Post-Biotics: Butyrate supplements and other postbiotics administered at specific times according to the circadian rhythm. Nurses oversee supplementation protocols using the digital twin, which provides dosing reminders and adherence tracking, while AI models forecast the best administration windows. A non-invasive neuromodulation method that can be used in nursing protocols is transcutaneous vagus nerve stimulation (tVNS). VNS devices modulate inflammatory tone and sleep architecture by stimulating the vagus nerve’s auricular branch. The digital twin gives real-time feedback on the effectiveness of tVNS sessions, which are started and monitored by nurses.

The gut-brain axis is essential to this ecosystem because new research shows how deeply the gut microbiota affects sleep, mental clarity, and emotional well-being. Emerging research indicates that gut dysbiosis—defined as an imbalance in gut microbial composition—is associated with three interconnected neuropsychiatric and sleep-related outcomes: mood fluctuations, cognitive impairment, and sleep disorders. Complementary interventions—specifically probiotics and postbiotics, which target gut microbiota composition—have demonstrated therapeutic potential for mitigating the effects of gut dysbiosis (54, 55). Within the digital-twin ecosystem, AI algorithms analyze two key datasets: gut microbiome profiles and sleep metrics derived from electroencephalography (EEG). These analyses identify correlations that inform the design of targeted probiotic interventions and personalized dietary regimens. When administered as postbiotics, SCFAs—a class of gut-derived metabolites—exert two beneficial effects: reducing neuroinflammation and improving cognitive function. These findings collectively highlight a promising opportunity: leveraging gut health to optimize three interconnected domains—sleep quality, cognitive function, and emotional well-being (54). Researchers can evaluate brain-heart interaction dynamics during sleep using two analytical techniques: time-delay analysis and delay-correlation landscapes (DCL). Furthermore, DCL specifically uncovers universal patterns that characterize physiological states and their transitions during sleep (42). Within the digital-twin ecosystem, this analytical approach yields insights into two critical areas: the neurophysiological regulation of sleep and the neurobiological underpinnings of cognitive-emotional health. These insights, in turn, enhance the accuracy and efficiency of targeted nursing interventions.

Within the digital-twin nursing ecosystem, the system gains value from the integration of contextually tailored Ecological Momentary Assessment (EMA) emotion labels—labels that provide real-time, patient-specific emotional data to inform care decisions. The EMA methods help in understanding the ups and downs of emotions by using self-reports at different times (56). This data is important as poor quality of sleep is associated with higher emotional reactivity and impairment of mood regulation especially when looked at the sleep microstructure (57). Studies have shown that the lack of restorative sleep can worsen an individual’s stress and anxiety (58). The innovations enabled by the project can lead to more targeted interventions to disordered sleep, such as digital cognitive behavioral therapy (CBT-I), or personalized sleep hygiene advice. This pattern recognition of emotional dysregulation and disordered sleep is made possible through the EMAs’ emotional data (59).

The ecosystem acknowledges that there may be clinically significant differences between objective physiological signals and subjective self-reports. The system uses a multimodal discrepancy resolution protocol that incorporates various data streams and contextual information instead of viewing such discrepancies as errors that need to be fixed. Each data stream is given context-dependent weights by the system using dynamic Bayesian networks. Since subjective experience is the main source of information during waking hours, EMA self-reports are given more weight. EEG-derived sleep staging is given more weight during sleep periods since it represents an objective neurophysiological state. The dynamic weighting model is updated on a regular basis according to temporal alignment and signal quality. The system initiates a verification cascade upon detecting a discrepancy. Autonomic verification: Wearable technology is used to measure heart rate variability (HRV). Somatic arousal that was not detected by EEG alone may be indicated by elevated sympathetic tone during the conflicting epoch. Micro-arousal detection: To find sub-cortical arousals that may accompany subjective anxiety but are not visible to standard sleep scoring, EEG microstructure is reanalyzed at finer temporal resolution. Multi-modal triangulation: To get a complete picture of the patient’s condition, actigraphy, environmental sensors, and other physiological streams are combined.

Based on the verification cascade, the explainable AI (XAI) layer produces comprehensible outputs. EMA reported anxiety at [time], but EEG shows N3 sleep, if HRV indicates an elevated sympathetic tone. HRV, on the other hand, shows sympathetic activation. Somatic arousal without cortical arousal could be the cause of the patient’s unconscious physical tension during deep sleep. EMA reports anxiety with concurrent micro-arousals” if micro-arousals are found. Sub-cortical awakenings—anxiety may be disrupting sleep at a level not detected by conventional macroarchitecture scoring—is one possible explanation. EMA anxiety reported without objective physiological correlates in the event that neither autonomic nor micro-arousal evidence is discovered. Misperception of the sleep state or a panic attack with low motor tone during the night are two possible interpretations.

The system produces an Ambiguity Alert when either modality’s confidence falls below 70%. instead of taking independent action. displays a dashboard with unified multi-modal evidence. By acknowledging that subjective–objective discrepancies are not mistakes but rather clinically instructive phenomena requiring human interpretation, this method guarantees that the system supports clinical judgment rather than replaces it.

Traditional emotion recognition methods often rely on subjective self-reports or physiological signals like EEG, which can be influenced by external factors (60). However, the EMA Emotion Label uses AI to evaluate multimodal data, such as physiological signals, voice intonation, and facial expressions, to provide a more thorough and accurate evaluation of emotional states (60). This is especially helpful in spotting minute emotional shifts that might point to underlying sleep or cognitive problems.

The digital-twin nursing ecosystem exhibits versatility across three key functional domains: personalized medicine, mental health care, and general healthcare delivery. Within the broader field of medicine, the ecosystem supports clinicians in two core clinical tasks: diagnosing and treating a range of disorders linked to the gut–brain–sleep axis. These disorders include emotional dysregulation disorders, cognitive impairments, and sleep disturbances (33, 51). In the domain of mental health care, the ecosystem offers novel insights into common psychiatric conditions—including anxiety disorders and major depressive disorder (MDD)—that are frequently associated with sleep disturbances and gut dysbiosis (28, 61). Within personalized medicine, the ecosystem facilitates the development of tailored therapeutic interventions—interventions that target the unique bidirectional connections between an individual’s gut microbiota, central nervous system (CNS), and sleep-related behaviors.

Digital cognitive tests hold notable importance in clinical and research settings, as they deliver effective, scalable cognitive assessment tools. In addition, they non-invasively assess the executive control, memory, attention, etc. The primary goal of these cognitive assessments is to detect early biomarkers of emotional distress or age-related cognitive decline. This early detection, in turn, enables clinicians to implement timely, targeted interventions. Integrating AI into these testing frameworks enables the development of adaptive testing protocols—protocols that dynamically adjust to an individual’s real-time performance. This adaptability ensures the delivery of precise, personalized cognitive evaluations. Beyond assessment, the digital-twin ecosystem can further correlate objective sleep data (e.g., sleep stage duration, wakefulness frequency) with cognitive test outcomes—supporting the exploration of potential causal relationships between sleep patterns and cognitive function. Targeted sleep interventions—such as sleep hygiene education or personalized sleep schedule adjustments—can directly address sleep-related issues like suboptimal sleep quality. Left unaddressed, this poor quality often manifests as measurable cognitive deficits, including memory lapses and attention impairments (62, 63).

Functional magnetic resonance imaging (fMRI) evidence demonstrates that sleep deprivation induces measurable reconfigurations of neural recruitment patterns within the hippocampo-cortical network—a network that supports declarative memory. This reconfiguration establishes a neurobiological signature of cognitive impairment directly attributable to sleep loss (64). Understanding the role of sleep in regulating cognitive circuit function is particularly relevant to the digital-twin ecosystem’s focus on sleep–cognition–emotion interactions. Notably, the fMRI and neuroimaging studies referenced above directly investigate this regulatory role of sleep. A multimodal neuroimaging approach maps metabolic oscillations across cortical energy landscapes while individuals engage in cognitive tasks. This mapping generates a real-time physiological substrate that supports two key objectives: digital phenotyping of brain function and continuous monitoring of dynamic brain states (65). Clinical cohort studies demonstrate that disruptions in sleep architecture are associated with two adverse outcomes in breast cancer survivors: increased objective cognitive decline and heightened affective distress. These findings further situate sleep–cognition interactions within the framework of the behavioral oncology model (66). The bidirectional links between emotional well-being, cognitive strategies, and sleep quality highlight the need for digital cognitive tests—tests designed to quantify these complex interactions within the digital-twin ecosystem’s defined objectives. Insights into cellular mechanisms of sleep regulation can inform the design of targeted digital cognitive tests—tests that align with the ecosystem’s focus on personalized, biology-driven assessments. A recent review delineates how thalamic reticular nucleus (TRN) spindle–slow oscillation coupling regulates two critical processes: gating synaptic plasticity during local sleep and calibrating hippocampo-cortical networks to optimize next-day cognitive performance (67). The interpretation of digital assessments can be improved by these insights into sleep regulation mechanisms, which establish a connection between neural circuit activity and cognitive outcomes and sleep quality. It turns out that the gut-brain axis is an important biological pathway that affects sleep and cognition. Evidence indicates that gut-microbiota-dependent modulation of serotonin and catecholamine signaling systematically tunes synaptic plasticity and executive performance, establishing microbial regulation of neurotransmission as a determinant of cognitive output (68). In older adults with insomnia, Haimov et al. further demonstrate links between gut microbiota composition, sleep quality, and cognition, indicating that microbiota profiles may function as biomarkers within the framework of the digital twin (69). There is potential for improving cognition through interventions that target the gut microbiota. According to Önning et al., taking probiotics (Lactiplantibacillus plantarum HEAL9) helps people who are under stress think better, suggesting that altering gut microbiota can affect cognitive processes related to sleep and emotional control (70). The role of the microbiota in sleep-related cognitive circuits is highlighted by Sun et al.’s demonstration that the probiotic strain BC99 reduces neuroinflammation and restores gut microbiota balance in mice that suffer from chronic sleep deprivation (71). Additionally pertinent are digital interventions like game-based cognitive training. Digital cognitive tests can be used as both assessment tools and therapeutic modalities within the ecosystem, as demonstrated by Yorozuya et al.’s findings that digital game interventions can enhance a variety of cognitive domains in older adults (72). All of these studies highlight how intricately sleep, gut microbiota, neural circuits, and cognition interact. In the AI-powered “gut–brain–sleep” ecosystem, they offer a scientific foundation for creating digital cognitive tests that represent the functional state of sleep-cognition-emotion circuits. This integration of biological, behavioral, and technological insights advances personalized nursing interventions.

### 3.4 Person–nurse–environment triad

A critical layer (Person-Nurse-Environment Triad) is Scenario Granularity, which secures high-resolution, context-specific data streams to render individual health trajectories with millisecond-to-millisecond and room-to-room fidelity. This layer incorporates various data streams, including genomics, metabolomics, and behavioral patterns, to create dynamic, real-time replicas of patients’ physiological states by utilizing digital twin technology (73). According to the tenets of precision medicine, this granular approach guarantees that interventions are customized to each patient’s particular biological and environmental circumstances (73).

The Observable Indicators and Sensor Matrix show cases the addition of multi-modal sensors to monitor key biomarkers and environmental parameters. Wearable technology, implantable sensors and IoT-enabled devices continuously collect data on gut microbiota, neural activity, and sleep quality to create an individualized health profile (73). The Connected framework explains that sophisticated data integration and standardization techniques are utilized to tackle the troubles of data fragmentation (74). The sensor matrix improves diagnostic accuracy and helps to detect irregularities as well as undertake preventive measures at an early stage (43). Biomarkers linked to the gut–brain axis—such as gut microbial metabolites or peripheral inflammatory cytokines—can be monitored in real time. This real-time monitoring supports the early prediction of neuroinflammatory responses, a key precursor to cognitive or emotional disturbances. The systematic analysis of sleep patterns—including metrics like sleep stage duration, wake-after-sleep-onset (WASO), and sleep efficiency—enables the early identification of sleep disturbances associated with age-related cognitive decline (73).

The sensor matrix includes features that allow for ongoing, non-invasive monitoring of microbial functional activity. VOC sensors built into smart toilet systems allow for real-time fecal headspace analysis. Exhaled microbial metabolites can be continuously detected with wearable breath analyzers. Rectal swab kits for patient-administered sampling in situations where collecting the entire stool is time-consuming. By capturing metabolite flux rates instead of static abundance, these devices minimize patient burden and provide high-temporal-resolution functional data. Every sensor stream is incorporated into the digital twin’s data processing pipeline after being time-stamped to correspond with circadian rhythms.

Non-invasive monitoring of microbial functional activity, the sensor matrix incorporates VOC sensors integrated into smart toilet systems and breath analyzers. These devices detect microbial metabolites—including SCFAs and tryptophan derived compounds in real time, capturing metabolite flux rates rather than static abundance. This approach reduces patient burden associated with daily stool sampling while providing high-temporal-resolution functional data for the digital twin model.

The Nursing Intervention Nodes layer within the digital-twin ecosystem translates data-driven insights into actionable, evidence-based clinical interventions. This layer leverages AI-powered clinical decision-support systems to recommend personalized interventions tailored to individual patient needs. These interventions may include targeted dietary adjustments and cognitive behavioral therapy for insomnia (CBT-I) (73). Deploying AI models within this layer ensures two critical attributes of interventions: their alignment with evidence-based practice and their adaptability to dynamic changes in a patient’s clinical condition. Furthermore, this layer fosters collaborative partnerships between patients and healthcare professionals—partnerships that empower individuals to engage actively in their own care management (74). Although sleep hygiene techniques can be modified to align with an individual’s circadian rhythms, AI-guided recommendations offer a more targeted approach: optimizing gut microbiome health through the administration of personalized probiotic regimens (73).

Critical indicators, including the microbial deficit risk score, sleep efficiency metrics, and active alert counts, are displayed as standardized zero-to-one-hundred composite scores rather than raw data streams. This abstraction eliminates the need for nurses to interpret complex multidimensional data in real time. Alerts are stratified into three categories: Green indicates autonomous system resolution with no nursing intervention required; Yellow denotes supervised intervention with algorithmic guidance; and Red signals urgent conditions demanding immediate nurse response. Only Yellow and Red alerts are surfaced to the nursing dashboard, filtering out noise and preserving attention for clinically significant events. Detailed physiological data, such as electroencephalographic spectrograms and raw microbiome abundance profiles, are accessible exclusively when composite scores exceed predefined clinical thresholds. This conditional access prevents information overload while ensuring that granular diagnostic information remains available for clinical decision-making when warranted.

Non-invasive therapies that fit the scope of nursing practice are given priority in the ecosystem. Circadian lighting optimization: AI-driven melanopic lux scheduling and customized light exposure schedules offered through IoT-enabled solutions.AI-guided insomnia treatment with nurse-in-the-loop assistance, including adherence tracking and escalation procedures, is known as digital CBT-I. Chronobiotic supplementation: Nurses using digital twin support provide prebiotics, probiotics, and postbiotics at circadian times. Environmental changes include aromatherapy, temperature adjustment, and noise control procedures. Nurses can use gentle touch, massage, and audio stimulation as sensory interventions at the patient’s bedside. The patient interface of the digital twin provides guided visualization, progressive muscular relaxation, and mindfulness as relaxation treatments. Personalized advice on sleep hygiene is given via the nurse interface, together with automated follow-up and adherence monitoring. The digital twin provides real-time feedback for all interventions, allowing nurses to evaluate their effectiveness and modify procedures.

To ensure clinical usability and address the complexity of AI-driven outputs, the ecosystem employs a nurse-in-the-loop (NIL) interface architecture specifically designed to abstract algorithmic complexity. This architecture translates raw multi-omics and EEG data into a three-tier alert system stratified by urgency and required nursing action: Green (Autonomous) for system-managed adjustments; Yellow (Supervised) for recommendations requiring nurse verification, accompanied by confidence intervals from uncertainty-aware models; and Red (Urgent) for immediate nursing intervention. All outputs are further converted into standardized nursing terminology compatible with NANDA-I clinical labels and presented via visual analytics—such as heatmaps for microbial risk trajectories—to enhance interpretability and reduce cognitive load.

Implementation of the ecosystem is supported by a structured hybrid training module (8-hour certification) comprising: (a) microbiome fundamentals for nursing practice, (b) interpretation of XAI-generated causal graphs, and (c) clinical escalation protocols for handling algorithm uncertainty. This training is delivered through an embedded simulation-based training module, where nurses can safely practice interpreting system outputs and delivering interventions in virtual patient scenarios prior to real-world deployment. This approach ensures that the technology complements—rather than overwhelms—clinical workflow and professional autonomy.

The “AI Modeling Challenges and Nursing Strategies” layer within the digital-twin ecosystem addresses two interconnected sets of issues: technical and ethical considerations inherent to AI implementation in healthcare settings. This layer specifically addresses key challenges—including AI model interpretability, algorithmic bias, and patient data privacy—that directly impact the trustworthiness and clinical utility of AI tools in nursing practice (75). The development of AI models characterized by openness, fairness, and patient-centeredness is enabled by a multidisciplinary approach that integrates design science principles and systems engineering methodologies (76). A core emphasis of this layer is the critical role of clinician—particularly nurse—engagement in AI model development. This involvement serves two key purposes: fostering trust among end-users (e.g., nurses, patients) and preserving the clinical relevance of AI tools (75). Hybrid AI models— which integrate deep learning architectures with traditional statistical techniques—enhance model interpretability. This approach addresses a key technical challenge by balancing the predictive power of deep learning with the transparency of conventional methods. Established ethical frameworks—such as principles of beneficence, non-maleficence, and respect for personhood—can support the protection of patient privacy and the preservation of patient autonomy in AI-driven healthcare (75).

By integrating two complementary pillars—cutting-edge health technology innovations and patient-centered care best practices—the digital-twin nursing ecosystem adopts a holistic, patient-focused approach to care delivery. The “gut–brain–sleep” digital-twin ecosystem—built on three core technologies: digital twin frameworks, artificial intelligence (AI), and multimodal sensing systems—provides a targeted solution for managing complex multi-system disorders. While the ecosystem delivers personalized benefits at the individual patient level—such as tailored treatments for gut, brain, or sleep issues—its application also holds broader implications for public health initiatives and population health management (73). Healthcare systems can leverage the ecosystem’s predictive models to optimize the allocation of limited clinical resources—such as staffing, diagnostic tools, or therapeutic interventions. Furthermore, aggregated ecosystem-wide data can be analyzed to inform the design of targeted public health interventions—specifically for conditions linked to sleep disturbances or gut dysbiosis (73). Additionally, by facilitating interdisciplinary collaboration in precision medicine and engaging patients as active partners in care co-creation, the ecosystem ensures that technology delivers tangible health outcomes (74).

A key innovation of this layer lies in its adaptability to evolving healthcare contexts—specifically, the diverse clinical scenarios associated with chronic conditions (e.g., type 2 diabetes mellitus), neurodegenerative disorders, and sleep-related illnesses. By merging heterogeneous data sources and supporting their interoperability with ontologies (77, 78). The system can assess physiological measurements and environmental parameters to provide personalized recommendations on sleep problems (79, 80). The predictive analytics in the scenario granularity layer supports the use of machine learning algorithms to foresee potential health risks such as the development of hepatic encephalopathy in a sick patient associated with gut dysbiosis and the impact of sleep deprivation on cognitive decline (33, 61). Integrating real-time optimizing generative AI models that model probabilities and fines of an uncertain future improves this accuracy (11, 81). The Layer within the digital-twin ecosystem delivers multiple distinct technological advantages tailored to advancing personalized healthcare. First, it advances patient-centric care by moving beyond a one-size-fits-all model—instead tailoring interventions to align with each patient’s unique health profile, which integrates data on gut microbiome composition, sleep patterns, and cognitive-emotional status (77, 78). It fosters interdisciplinary collaboration and cross-referencing of datasets from complementary fields—including genomics, neuroscience, and behavioral science—to deepen understanding of the gut–brain–sleep axis (29, 82). Third, the layer ensures scalability by harnessing cloud and edge computing infrastructure to process large volumes of real-time multimodal data with high efficiency (83). By prioritizing semantic accuracy and rigorous data quality assurance, the layer guarantees that digital twin outputs are both reliable and actionable for clinical decision-making (61).

The Scenario Granularity Layer within the digital-twin ecosystem demonstrates clear potential application value for advancing personalized healthcare transformation. Specifically, this layer can support the development of personalized therapeutic strategies for neurodevelopmental disorders—such as autism spectrum disorder (ASD)—which frequently present with comorbid sleep disturbances and gut microbiota dysbiosis (61). he layer exhibits utility for managing chronic conditions—including type 2 diabetes mellitus (T2DM)—where the complex interplay between metabolic processes, gut health, and sleep quality exerts a meaningful impact on disease progression (77). A core mechanism of the Scenario Granularity Layer involves modeling and predicting the outcomes of targeted interventions—including dietary modifications or fecal microbiota transplantation (FMT). This capability drives a paradigm shift from reactive to preventive care within healthcare systems (11, 33).

## 4 XAI-driven clinical engines

### 4.1 Multi-omics & EEG representation learning

At the core of the gut–brain–sleep digital-twin ecosystem lies representation learning—a foundational machine learning technique that facilitates the discovery of meaningful, task-relevant features. This technique is especially valuable for analyzing complex datasets—such as those derived from sleep monitoring devices, neuroimaging, and gut microbiota sequencing—which frequently exhibit intricate interdependencies and nonlinear relationships (84, 85). Specifically, representation learning enables two key analytical capabilities: forecasting changes in gut–brain axis communication triggered by dietary modifications and identifying microbial signatures associated with sleep disorders. By converting raw, unstructured data into interpretable feature representations, this technique enhances the accuracy and efficiency of predictive models. These models, in turn, support the identification of clinically relevant biomarkers and therapeutic targets (84, 86). Complementing these analytical capabilities, digital twin technology enables the development of patient-specific virtual representations—digital replicas that mirror the unique physiological states and dynamic health trajectories of individual patients. These can be updated in real time with data to model the outlook for health outcomes (87, 88). This ability is particularly important for personalized medicine as it allows doctors to customize actions to each patient’s gut, brain, and sleep ecosystem.

The digital twin framework is not merely a model. It is a system that changes as new information changes. By adopting an incremental learning strategy like the Broad Learning System (BLS), the system can adapt to changes in the patient’s condition without heavy retraining. The impact of chronic conditions can be mitigated by continual monitoring and intervention. Denoising autoencoders (DAEs)—a class of unsupervised learning models—enhance the performance of feature extractors by mitigating noise and handling data incompleteness. In the context of the gut-brain-sleep digital twin ecosystem, DAEs are specifically applied to filter physiological noise from EEG signals and biological noise from microbiome sequencing data. This automated noise filtering ensures that downstream predictive models operate on high-quality signals, and that the clinical outputs presented to nurses are free from spurious fluctuations. This capability makes DAEs particularly applicable in healthcare settings, where noisy or incomplete datasets are prevalent (43, 87). Within the patient-specific gut–brain–sleep digital-twin ecosystem, the DAE-BLS hybrid model demonstrates the synergistic integration of DAEs and broad learning systems (BLS) for two key tasks: extracting high-quality multimodal features and forecasting diseases with robust accuracy (43). To address the challenge of functional redundancy—where different microbial taxa produce identical metabolites—the ecosystem employs PICRUSt2 (Phylogenetic Investigation of Communities by Reconstruction of Unobserved States) to predict metagenomic functional pathways from 16S rRNA sequencing data. These predicted pathways are combined with direct metabolomic measurements to create a functional redundancy-corrected feature space. This approach reduces noise arising from taxonomic variability and ensures that predictive models focus on functional output rather than taxonomic composition alone. Denoising autoencoders are employed to eliminate motion artifacts and electromagnetic interference. These models are trained to reconstruct clean electroencephalographic signals from corrupted inputs, with explicit constraints to preserve sleep-relevant neurophysiological features including sleep spindles and slow oscillations. Bayesian outlier detection algorithms identify and filter contamination spikes arising from dietary confounders and batch effects. Taxa present at abundances below the detection threshold are excluded from subsequent analyses to prevent spurious correlations. The system implements uncertainty-aware sampling protocols. When variance in microbiome composition falls below predefined thresholds, sampling frequency is automatically reduced, for instance, from every seventy-two hours to weekly intervals in cases of microbiome stability. This approach reduces data volume without compromising predictive accuracy.

One of the most innovative capabilities of the gut–brain–sleep digital-twin ecosystem is its ability to simulate and predict the effects of interventions on the gut–brain–sleep axis. The ecosystem can model the impact of interventions such as probiotic supplementation, dietary adjustments, or sleep hygiene protocols on three key components: gut microbiome composition, neural activity patterns, and sleep quality metrics. Knowledge graphs facilitate two critical knowledge-management tasks: converting current scientific literature and findings into machine-readable formats, and enabling the identification of causal relationships and mechanistic biological pathways (85, 87). By integrating multiomic data into these knowledge graphs, the ecosystem can generate novel insights into the gut–brain–sleep axis—such as the influence of gut-derived hormones on sleep architecture or the role of specific microbial metabolites in circadian rhythm regulation (85, 89). A comprehensive understanding of these insights can inform the development of targeted therapeutic strategies for diverse sleep disorders and their comorbidities—including interventions such as neuromodulation or microbiome-targeted therapies. The ecosystem’s purpose extends beyond individual-level care to include population-level research—specifically, monitoring interactions between the gut, brain, and sleep, as well as tracking their trends and patterns across diverse demographic groups. The ecosystem can investigate how environmental exposures, lifestyle factors, or aging processes influence gut microbiota composition and sleep quality—with findings that hold implications for designing evidence-based public health campaigns. Also, if reinforcement learning is used, the ecosystem will be trained to use only the most effective interventions, and future recommendations will be optimized (45, 90). The process makes the ecosystem responsive to newer data and enhances its usability for clinical and research applications. According to several research studies, neural reorganization that hinges on sleep is vital for the advancement of motor skills following engaging a certain skill.

Studies of various processes that are linked to sleep indicate how important sleep is for enhancing motor memories (91). As AI models are able to replicate sleep-related neural plasticity, they can improve motor learning and recovery in the case of digital twins. Gut microbiota’s impact on sleep and brain function has gained much attention as in which MGBA mediates a variety of physiological and behavioral phenomena, research show (3, 8). Essentially, alterations in the gut microbiota and microbiome have been linked with sleep disorders as well as neurological and psychiatric disorders. Therefore, microbiota-targeted interventions may be therapeutic approaches to improve sleep and cognitive function (8, 92).

Recent studies show that we can model and study their relations using AI. BrainCog and other similar brain-inspired artificial intelligence (AI) designs make it increasingly simple to simulate brain activity. Designs like these could be incorporated into digital twins to imitate brain-like neural responses and plasticity (93). The use of machine learning techniques on deep brain signals can help in accurate classification of sleep stages that could help design personalized sleep management programs. For the gut-brain-sleep ecosystem, the development and application of a digital twin remain essential. This digital twin should integrate multiple complementary technologies—specifically, blockchain, edge computing, and secure communication protocols (94). With this integrated digital twin system in place, researchers or clinicians can monitor and simulate an individual’s physiological states in real-time. This real-time monitoring and simulation, in turn, creates conditions for implementing tailored interventions—interventions that are closely dependent on the individual’s specific neural activity, sleep patterns, and gut microbiota composition. A critical next step involves two key tasks: first, the clinical validation of the aforementioned digital twin system; second, the creation of targeted microbiota modulation strategies—strategies designed to specifically improve sleep quality and cognitive functions. As recent review studies have pointed out, AI holds significant potential to support the development of personalized therapeutics, particularly those targeting the gut microbiota to enhance sleep regulation and cognitive performance (8, 95). Specifically, strategies that leverage AI-based predictive modeling may offer a viable approach to proactively address certain disorders—for instance, neuropsychiatric disorders and sleep-related disorders. This proactive intervention can be achieved by optimizing neural plasticity and modifying the composition or activity of the gut microbiota.

### 4.2 Counterfactual digital-twin causality

Research under the framework of the Microbiota-Gut-Brain Axis (MGBA) has demonstrated that the gut microbiome exerts regulatory effects on sleep patterns and emotional health. This regulatory role, as noted, is mediated through complex interactive processes among distinct microbial ecosystems within the gut (96). In recent empirical studies, researchers have utilized the Pittsburgh Sleep Quality Index (PSQI)—a widely accepted tool for quantifying sleep quality in clinical and research settings—to assess sleep parameters. These studies have drawn the conclusion that an individual’s sleep quality exhibits a strong correlation with two key gut microbial features: first, the overall diversity of the gut microbiota; second, elevated levels of specific bacterial genera, including Blautia, Ruminococcus, and Prevotella (97). The collective findings from these and related studies further suggest that certain microbial metabolites—most notably butyrate and SCFAs—play an essential role in regulating three interconnected physiological processes: brain activity, circadian rhythmicity, and sleep architecture (3, 98). Animal model studies—specifically those conducted in laboratory rodents, a common model for sleep and microbiome research—have shown that butyrate-producing bacterial families, namely Lachnospiraceae and Ruminococcaceae, possess the ability to enhance non-rapid-eye movement sleep (NREMS) (99, 100). Beyond its effects on metabolites and sleep structure, the gut microbiota also acts on two key biological pathways that influence mood and sleep. On one hand, it modulates inflammatory signaling pathways; on the other hand, it interacts with neurotransmitter systems—including those involving serotonin and γ-aminobutyric acid (GABA)—both of which are well-documented regulators of mood stability and sleep homeostasis (101, 102).

The biological insights generated within the digital-twin ecosystem—specifically via AI-driven computational models—enable the development of real-time, personalized simulations. These simulations focus on an individual’s unique gut-brain-sleep axis, capturing dynamic interactions between its three core components. To achieve these simulations, the digital-twin ecosystem analyzes multi-modal data streams: wearable device metrics, gut microbiome sequencing data, and standardized behavioral assessments. Leveraging this integrated data, the ecosystem not only predicts fluctuations in sleep quality and emotional well-being but also supports the optimization of these outcomes through targeted microbiome interventions (103, 104). Within this ecosystem, AI systems play a key role in identifying specific microbial taxa that correlate with mood disturbances, emotional dysregulation, and sleep disorders. Based on these correlations, the systems can generate evidence-informed recommendations: these may include the administration of targeted probiotic supplements or personalized adjustments to dietary patterns—interventions designed to restore microbial balance and alleviate symptoms (96, 105). This integrated approach—combining digital simulation, AI analysis, and microbiome interventions—holds significant therapeutic potential for conditions characterized by both sleep disturbances and gut microbial abnormalities. A prime example is insomnia, which research has linked to reduced gut microbial alpha diversity and gut dysbiosis (106, 107). Another key strength of the digital-twin ecosystem lies in its capacity to simulate circadian rhythms—the 24-hour biological cycles that regulate sleep-wake patterns and metabolic function. Researchers are now harnessing this simulation capacity, alongside an understanding of how circadian rhythms interact with the gut microbiome, to develop novel therapeutic strategies. These strategies target not only sleep disorders but also metabolic diseases, such as type 2 diabetes mellitus and obesity—conditions increasingly linked to circadian-microbial dysregulation (108, 109).

A key technological strength of this specific ecosystem lies in the integrated merging of multi-omics datasets—including transcriptomics, metabolomics, and metagenomics—with the primary goal of achieving a holistic, system-level understanding of the gut-brain-sleep axis (110). By integrating these diverse omics data streams—transcriptomics, metabolomics, and metagenomics included—it further enables the delivery of a more comprehensive, integrated insight into the functional mechanisms of the gut-brain-sleep axis. Machine learning algorithms, for their part, contribute to the identification of novel biomarkers and potential treatment targets: their unique capacity to detect subtle patterns and hidden correlations often eludes conventional analytical techniques.

The ecosystem uses a Heterogeneous Graph Neural Network with Counterfactual Attention (HGNN-CF) as the main Layer 2 engine to enable transparent and causal modeling of the gut-brain-sleep axis. The three types of nodes that make up the ecosystem’s core layers are used to create a heterogeneous knowledge graph:

Individual microbial taxa and their useful metabolites are known as microbial nodes. Immune nodes include inflammatory signaling intermediates, microglial activation states, and cytokine profiles. Neural nodes include neuroinflammatory markers, vagal tone metrics, and sleep stages. The model is able to capture both established mechanisms and new associations because edges are weighted by a combination of learned statistical correlations from patient data and known biological pathways. The HGNN-CF integrates Counterfactual Graph Attention Networks (CF-GAT) to facilitate causal inference and “what-if” scenario modeling. For each perturbation, the CF-GAT computes the factual prediction, which represents the projected neuro-immune outcome under current conditions.

The anticipated result under the disturbed condition is known as a counterfactual prediction. Each microbial node and edge’s contribution to the anticipated change is represented by an attribution score. By enabling nurses to model intervention effects prior to implementation, this capability aids in clinical decision-making. The HGNN-CF creates attention weights that are displayed as neuro-immune pathway heatmaps in the nurse interface to make sure Layer 2 is not a “black box.” The heatmap delineates, for a specific prediction, the primary microbial taxa contributing to activation, the intermediary immune nodes responsible for signal propagation, and the neural consequences most substantially modulated.

### 4.3 Uncertainty-aware XAI

Specifically, it guarantees that the predictions and recommendations generated by AI models remain interpretable and transparent to healthcare professionals. However, they are not very transparent. Lack of transparency hurts confidence and limits clinical application (115, 116). By infusing the XAI4Diabetes framework into the digital twin ecosystem, that adopts a comprehensive approach, we can bridge this gap that offers interpretable insights into the decision-making process according to its data inputs and model outputs (115). Particularly, interventions that might be effective in this area require knowledge of the relevant interactions and mechanistic pathways of gut microbes (11, 28). Custom-made treatment strategies are possible because of explainable models that explain how certain microbial taxa or metabolites affect cognitive function or sleep quality (28, 65).

Microbiomes deal with problems that can be analyzed in a way that improves their robustness. These problems involve using severe variable and noise. Machine learning models that are trained on sparse data and high dimensional space are often not very accurate, as they are unable to generalize outside the training data (11, 117). The digital twin ecosystem incorporates uncertainty metrics that give confidence intervals on the model outputs. This allows the clinicians to assess the correctness of the model outputs and determine their decisions wisely (117). In addition, the ecosystem translates these uncertainty estimates into nurse-friendly formats, such as visual confidence bands accompanying risk scores, enabling nurses to gauge the reliability of each recommendation without needing to interpret raw probabilistic outputs. The ecosystem prioritizes metabolite-based features which exhibit lower technical noise and higher biological stability compared to taxonomic features. This prioritization improves model robustness and clinical interpretability, as metabolite concentrations are less susceptible to transient fluctuations in low-abundance taxa. This is particularly important in precision medicine, where customized interventions must take into account the unique microbiome and clinical profile of each patient (65, 117). Models that take uncertainty into account can identify individuals for early microbial transplant or dietary change intervention but still limit harm from spurious intervention (11, 117).

It is used in clinical and research settings as well as for personal health management. The digital twin approach may add to current datasets through the generation of synthetic data resembling real patients. This enables the exploration of imagined scenarios and enhances the prediction precision of machine learning applications (117). This is particularly beneficial for studies examining the interface between the gut-brain-sleep axis, where there is little information about how microbiomes, neural signals, and sleep work together (28, 65). Due to the modular design of the ecosystem, the inclusion of other omics layers, such as metabolomics and epigenomics, allows for a more complete understanding of the biological pathways involved (65, 118).

Toh et al. refer to the gut microbiota as a dynamic component of the gut-brain interface. The gut microbiota may be important as an ecosystem that affects the brain and behavior as demonstrated by the possible role of gut microbiota in autism spectrum disorder (3). The relationship between gut microbiome diversity and sleep physiology is examined by Smith et al. They find that sleep deprivation can change the composition of the gut microbiota, though the results vary, with some studies showing no change (4). This ambiguity calls for additional research on the microbiome’s sensitivity to sleep states, using techniques like actigraphy in conjunction with microbiome sampling to elucidate these connections. Using molecular pathways that mediate microbiome-sleep interactions, Sen et al. explore the ways in which the microbiota-gut-brain axis affects sleep (6). They highlight how a number of variables, such as immune responses and microbial metabolites, can affect how well people sleep, demonstrating both the direct and indirect effects of microbiota on sleep architecture.

A longitudinal infancy cohort study demonstrates that emergent gut microbial α-diversity covaries with structural and functional brain maturation across the first postnatal year, thereby imprinting enduring signatures on subsequent sleep–wake architecture and establishing early-life microbe–brain crosstalk as a developmental determinant of sleep trajectories (119). According to Sgro et al., who investigate how the brain-gut-immune axis coordinates the development of the sleep-wake cycle throughout life, sleep or microbiota dysfunction can have a detrimental impact on general health and system integration (120).

The uncertainty-aware XAI framework has been extended to explicitly address discrepancies between subjective self-reports and objective physiological signals. For each multimodal data point, the system executes the following sequence: it computes confidence scores for each individual data stream, performs temporal alignment with state-level ecological momentary assessment reports, and applies dynamic Bayesian networks to assign context-dependent weights to the respective modalities.

To mitigate the risk of alert fatigue among nursing personnel, the ecosystem implements a multi-tier alert hierarchy. At the edge layer, artificial intelligence preprocessing handles ninety-five percent of data locally on wearable and Internet-of-Things devices. Only anomaly summaries are subsequently transmitted to central servers, thereby minimizing bandwidth requirements and server-side computational load. Federated learning aggregation performs population-level noise cancellation prior to the generation of individual alerts. This approach filters out systematic artifacts that might otherwise trigger false-positive notifications. The resulting alert rate is constrained to a maximum of zero-point-five percent of total data streams. This stringent threshold ensures that all nurse-visible notifications are clinically meaningful and immediately actionable. The nursing dashboard displays Composite Risk Scores on a standardized zero-to-one-hundred scale rather than raw data streams. Drill-down functionality to underlying physiological or microbiome data is enabled exclusively when scores exceed predefined clinical thresholds. This design permits nurses to access granular information only when such detail is warranted by clinical necessity.

When discordance between subjective and objective modalities exceeds a 70% confidence threshold, the framework generates an interpretable conflict summary. This summary integrates all available evidence, furnishes nursing staff with probabilistic interpretations of the likely underlying clinical phenomenon, and includes confidence intervals for each interpretation. Concurrently, the system triggers an Ambiguity Alert whenever confidence in either modality falls below 70%, thereby recommending nurse-led clinical assessment. This extension ensures transparency in instances where AI-generated predictions may appear counterintuitive to clinicians, thereby supporting—rather than supplanting—human clinical judgment.

Hepp et al. examine explainability and uncertainty in deep learning models that analyze brain MRI data for age estimation in the context of aging (121). Although it is not specifically addressed in their work, their method of interpreting physiological patterns—which includes uncertainty estimation and visual explanations—could be expanded to comprehend the aging-related alterations in the gut-brain-sleep axis. Han et al. and Wang et al.’s recent reviews summarize the data showing that sleep and gut microbiota communicate in both directions and highlight how microbiota-targeted therapies can enhance the quality of sleep (7, 8). Wang et al. describe the metabolic, immunological, and neural pathways by which the microbiota affects sleep and neuropsychiatric disorders, while Han et al. specifically highlight therapeutic approaches involving microbiota manipulation (7, 8).

By investigating how traditional Chinese medicine (TCM) can alter gut microbiota to treat insomnia, Feng et al. deepen our understanding of this topic and propose that microbiota regulation is a promising treatment option for sleep disorders (122). Naufel et al. also address the wider implications of the brain-gut-microbiota axis in the treatment of mental and neurological conditions, highlighting the potential of interventions that target the microbiota within this ecosystem (9). For the “gut–brain–sleep” ecosystem to advance individualized and dependable interventions, explainability and uncertainty estimation must be integrated when modeling these interactions (121). The entire body of work presented in this study aligns with the recommendations proposed by Alex and Pomp—specifically, their emphasis on developing interpretable and transparent analytical models. By leveraging these interpretable and transparent models, researchers can more effectively harness the regulatory influence of the gut microbiota on sleep physiology and quality.

### 4.4 Federated-edge continual learning

The integration of brain-inspired AI frameworks and spiking neural networks (SNNs)—which closely mimic the efficiency and adaptive plasticity of biological neural systems—also aligns effectively with the “gut-brain-sleep” digital twin ecosystem developed in this study (111, 112). Leveraging these neuromorphic computing models—paired with federated learning protocols—the “gut-brain-sleep” ecosystem achieves low energy consumption and reduced computational overhead, enabling efficient processing of high-dimensional, multimodal data streams (113). The BrainCog platform serves as a robust foundational tool for simulating core brain cognitive functions—specifically those implicated in the regulation of gut health and sleep-wake cycles. Critically, this platform also facilitates the design and validation of SNN-based AI models tailored to gut-brain-sleep interaction research (114). By enabling key applications—including biomarker identification, disease risk prediction, and the customization of patient-specific therapeutic strategies—the integration of AI and digital twin technology helps address the translational gap between preclinical research and clinical practice (115).

One of the most novel aspects of this ecosystem is the ability to leverage federated learning to mitigate data heterogeneity and interoperability issues. This framework ensures that the digital twin is sufficiently flexible to respond to personalized and population-level data by enabling the collaborative training of models on diverse data sets, such as gut microbiome profiles, brain imaging data and sleep data. Federated learning also minimizes catastrophic forgetting. It is a common result of regular deep learning models. It achieves this by allowing knowledge bases to be updated in pieces. There is no need for system retraining. This feature is particularly useful for the discussion of the “gut–brain–sleep” axis, given that ongoing model improvements and long-term data capture are needed to monitor these interactions. Recent studies show how edge AI and federated learning (FL) can enable decentralized, privacy-preserving data processing for a variety of applications. This is particularly critical for sensitive biosystems and health information. The work of Li et al. highlights the importance of mobile/multi-access edge computing (MEC) servers with AI in vehicular edge networks. They demonstrate how the application of AI can help in intelligent data sharing and service provisioning at the network edge (116). This strategy is an example of how edge AI can be used more broadly to manage dispersed data sources; this idea can be applied to ecosystems related to health, such as the gut-brain-sleep axis.

Edge-computing filters are used in the suggested ecosystem to pre-process data from non-invasive monitoring devices, such as wearable breath analyzers, smart toilet VOC sensors, and rectal swab data streams. These edge-based elements carry out: Sensor outputs are calibrated in real time to guarantee accuracy across devices. automated noise reduction with lightweight denoising algorithms customized for every kind of sensor. calculations of initial metabolite concentrations prior to transmission to central servers. Multi-modal data streams are temporally aligned with circadian timestamps. This edge-based preprocessing guarantees that subsequent predictive models receive high-quality functional data streams while minimizing latency and bandwidth requirements.

Within the proposed ecosystem, edge-computing filters are also deployed to pre-process raw multi-omics and EEG data at the point of collection. These filters perform three critical functions: (1) automated noise reduction using lightweight denoising algorithms; (2) imputation of missing data points based on temporal continuity; and (3) preliminary stratification of outputs into the three-tier alert system (Green/Yellow/Red) before transmission to central servers. This edge-based preprocessing minimizes latency, reduces bandwidth requirements, and ensures that nurses receive only curated, actionable summaries rather than raw data streams.

In their investigation of federated learning in healthcare, Gupta et al. suggest a hierarchical FL framework for anomaly detection in remote patient monitoring that makes use of digital twins (DTs) and edge cloudlets (117). In line with the requirements of sensitive biological data involved in gut-brain-sleep studies, their work highlights the significance of local data processing to reduce privacy concerns and security threats. Han et al. critically analyze the relationship between sleep and gut microbiota (GM), clarifying the feedback loop in the brain-gut axis and the potential benefits of microbiota modifications for enhancing sleep quality (7). This biological realization emphasizes the need for advanced, privacy-preserving data analysis frameworks that can manage sensitive and diverse data, which federated learning can offer. Yang et al. present advances in federated neuromorphic learning, introducing energy-efficient, brain-inspired models that allow edge devices to cooperatively train global models while maintaining privacy (118). Such neuromorphic approaches could be instrumental in developing low-power, real-time monitoring systems within the gut–brain–sleep ecosystem. The integration of blockchain with federated learning for secure IoT feature analysis is discussed by Alghamdi et al., illustrating how blockchain can enhance data security and trustworthiness in distributed AI systems (119). This is particularly relevant for health ecosystems where data integrity and privacy are paramount.

Within the proposed ecosystem, edge-computing filters are deployed to pre-process raw data from VOC sensors, breath analyzers, and other non-invasive monitoring devices. These filters perform real-time calibration, noise reduction, and preliminary metabolite concentration calculations before transmitting data to central servers. This edge-based preprocessing ensures that metabolite flux rates are accurately captured and that downstream models receive high-quality functional data streams.

In their review of privacy-preserving FL applications in healthcare, Moon et al. highlight how FL’s decentralized architecture can safeguard patient data while facilitating cooperative model training (120). As an example of the potential for federated models in neurobiological and sleep-related research, Viet et al. show how effective federated deep learning is at classifying brain tumors, achieving high accuracy without sharing sensitive data (121).

In their survey of federated learning’s function in the metaverse, Yenduri et al. highlight enabling technologies like blockchain and digital twins that can be modified to produce thorough, privacy-conscious digital representations of the gut, brain, and sleep system (122). In order to address complex, multi-modal biological ecosystems, their discussion emphasizes the significance of integrating AI, edge computing, and digital twin technologies.

Li et al. suggest federated learning driven by generative AI to get around resource constraints and data heterogeneity in mobile edge devices (123). Their “Filling the Missing” (FIMI) data approach can be especially helpful when modeling the gut-brain-sleep interactions, where variability and data scarcity are frequent problems. Himeur et al. examine the Internet of Energy’s edge AI landscape in general, offering insights into the difficulties and potential future developments of network edge AI deployment (124). Their analysis provides insightful recommendations for creating scalable, robust AI-powered ecosystems for health applications, such as the gut-brain-sleep axis. Federated and edge learning frameworks, enhanced by blockchain and digital twin technologies, have great potential for creating “gut–brain–sleep” digital-twin ecosystems that are intelligent, scalable, and privacy-preserving. By enabling three core functionalities—decentralized data analytics, enhanced data security protocols, and real-time monitoring of physiological health status—these neuromorphic and digital twin techniques facilitate the development of innovative, targeted interventions for personalized healthcare delivery, along with related translational applications.

### 4.5 Evidence tiering of technologies

#### 4.5.1 Existing supporting evidence

The emerging concept of an AI-empowered “gut-brain-sleep” digital-twin nursing ecosystem represents a transformative paradigm in precision medicine, integrating validated technologies to model and optimize individualized health outcomes. This ecosystem leverages time-series Transformers for sleep microstructure analysis, a method already demonstrated to enhance the classification of sleep stages and disorders by capturing temporal dependencies in polysomnography (PSG) data (125). Such AI-driven approaches are particularly suited for sleep medicine, where massive electrophysiological datasets require sophisticated pattern recognition to distill actionable insights (125). By combining PSG with multi-omics data, the ecosystem can uncover bidirectional relationships between sleep quality, microbial composition, and neurological function, as evidenced by studies linking gut dysbiosis to neuropsychiatric disorders via microglial modulation (28, 29).

A critical innovation lies in the digital twin framework, which synthesizes patient-specific data into dynamic, predictive models. The ecosystem could integrate mesoscale brain simulators, validated in electrocorticogram (ECO) studies, to model real-time neural activity and predict responses to interventions like neuromodulation or dietary changes (126). These simulators, powered by variational Bayesian recurrent neural networks, dynamically adapt to latent brain states, enabling virtual drug testing and functional network analysis (126).

The gut-brain axis is a focal point for this ecosystem, with microbial metabolites implicated in hepatic encephalopathy and cognitive decline (33). AI can map these interactions by analyzing metagenomic data to identify neurotoxic bacterial pathways, offering targets for therapeutic modulation. Targeting phenylalanine decarboxylase (PDC) in cirrhotic mice reversed neurological symptoms, highlighting the potential for microbiome-driven interventions (33). Similarly, vagal sensory neurons, which detect intestinal fat via specific receptors, provide a mechanistic link between gut stimuli and brain reward circuits (63). This aligns with the ecosystem’s goal of decoding interoceptive signals through neuroepithelial interfaces, where enteroendocrine cells paracrinally activate afferent nerves to influence behavior (127).

Evidence tiering ensures clinical translatability by prioritizing validated methods. For sleep analysis, AI models like CNNs and RNNs have achieved expert-level agreement in automated sleep staging, reducing reliance on manual scoring (125). Transfer learning further enhances these models, enabling cross-subject generalization with minimal labeled data (128). In gut-brain research, federated learning frameworks address data privacy concerns while pooling multi-institutional datasets to improve predictive robustness (129). The ecosystem also benefits from synthetic data generation, as demonstrated by GANs augmenting EEG datasets for emotion recognition and seizure detection, mitigating small-sample limitations (128).

Challenges include scaling digital twins to high-dimensional datasets and ensuring ethical data use. Hybrid approaches combining mechanistic models with AI-driven synthetic data may bridge this gap (33). Future directions involve closed-loop systems where real-time biomarker feedback refines digital twins iteratively, akin to industrial predictive maintenance (130, 131). By unifying these technologies, the ecosystem could revolutionize nursing care, offering proactive, personalized interventions for conditions like insomnia, cirrhosis-related encephalopathy, or microbiota-driven neuroinflammation (28, 33).

In conclusion, the AI-empowered gut-brain-sleep digital-twin ecosystem synthesizes cutting-edge computational tools with clinical insights to address complex, multi-system disorders. Its foundation in validated technologies—from time-series AI to microbiota modulation—ensures both innovation and reliability, paving the way for a new era of precision nursing. Future work should focus on interoperability standards, ethical AI governance, and translational trials to validate ecosystem efficacy across diverse populations (44, 129).

#### 4.5.2 Recent feasible evidence

The emerging concept of an AI-empowered “gut–brain–sleep” digital-twin nursing ecosystem represents a transformative frontier in personalized healthcare, leveraging cutting-edge technologies such as federated learning (FL) and edge computing to address the intricate interplay between gastrointestinal health, neurological function, and sleep regulation. This integrative approach aligns with the growing emphasis on precision medicine, where multi-omics data and AI-driven models are harnessed to tailor interventions to individual patient profiles (132). The digital twin framework, originally rooted in industrial applications, is now being adapted to biomedicine, offering a dynamic virtual representation of patient-specific physiological states. By simulating real-time interactions between the gut microbiome, neural pathways, and sleep patterns, this ecosystem could enable early detection of dysbiosis-linked neurological disorders or sleep disturbances, while optimizing therapeutic strategies through predictive analytics (39).

A cornerstone of this ecosystem is federated learning, which addresses critical challenges in healthcare data privacy and siloed datasets (133, 134). FL enables collaborative model training across decentralized institutions without raw data exchange, ensuring compliance with regulations like the GDPR. FL has been successfully integrated with Graph Neural Networks (GNNs) to classify diseases using Protein-Protein Interaction (PPI) networks, where human experts refine subgraph weights to enhance interpretability and accuracy. This methodology could be extended to the “gut–brain–sleep” axis, where FL aggregates heterogeneous data from wearable devices, electronic health records (EHRs), and multi-omics assays to train robust AI models while preserving patient confidentiality. Furthermore, edge computing complements FL by processing data locally on IoT-enabled devices, reducing latency and bandwidth constraints. Recent advancements in lightweight deep learning models, such as quantized neural networks, have made edge-based inference feasible for real-time monitoring of biomarkers like cortisol levels or gut-derived metabolites (135, 136).

The innovative synergy of these technologies lies in their ability to create a closed-loop feedback system. Edge devices could detect aberrant sleep patterns via accelerometer data, triggering federated AI models to analyze correlated gut microbiome shifts. Clinicians could then receive actionable insights through explainable AI (XAI) interfaces, which highlight causal relationships between microbial taxa and neuroinflammatory markers. Such a system aligns with the “virtual placebo” concept proposed for neurodegenerative trials, where digital twins simulate untreated disease progression to benchmark treatment efficacy (39). Additionally, synthetic data generation mitigates limitations of small datasets, as demonstrated in cardiac disease prediction, where synthetic twins improved model performance by emulating unobserved patient variants.

Despite its promise, this ecosystem faces implementation hurdles. Biophysical complexities of the gut-brain axis demand high-fidelity modeling, yet current digital twins often rely on imperfect approximations due to gaps in mechanistic understanding. Ethical concerns, such as algorithmic bias in FL models or data security risks in edge networks, necessitate robust governance frameworks (137, 138). Moreover, interoperability between EHRs, IoT devices, and AI platforms remains a technical challenge, though initiatives like the Personal Health Train offer federated solutions. Future research must prioritize validation studies across diverse cohorts to ensure generalizability, alongside the development of hybrid FL-edge architectures that balance computational efficiency with clinical accuracy (139).

The “gut–brain–sleep” digital-twin ecosystem epitomizes the convergence of AI, federated systems, and edge computing in nursing and preventive care. By harnessing FL for privacy-preserving collaboration and edge technologies for real-time responsiveness, this paradigm could revolutionize the management of complex, multi-system conditions. Its plausible near-term feasibility is underscored by existing successes in synthetic data augmentation, federated GNNs, and IoT-driven health monitoring (140). As the field advances, interdisciplinary collaboration—spanning clinicians, data engineers, and ethicists—will be pivotal to translating this vision into clinical practice, ultimately fostering a new era of patient-centric, AI-augmented healthcare.

#### 4.5.3 Long term speculative evidence

The emerging concept of an AI-empowered “gut–brain–sleep” digital-twin nursing ecosystem represents a speculative yet transformative frontier in personalized healthcare, integrating cutting-edge advancements in neurogastroenterology, computational modeling, and artificial intelligence. This paradigm envisions a dynamic, bidirectional feedback loop where real-time data from the gut microbiota, neural activity, and sleep patterns are synthesized into a high-fidelity digital twin—a virtual replica of an individual’s physiological state—to predict, prevent, and treat disorders spanning metabolic, neurological, and circadian domains (28, 29). At its core, this ecosystem leverages the gut-brain axis, a well-established communication network where microbial metabolites modulate neuroinflammation, neurotransmitter synthesis, and even behavior (28, 33). Dysbiosis-driven production of phenylethylamine by Ruminococcus gnavus has been linked to hepatic encephalopathy via impaired monoamine oxidase-B activity, illustrating the microbiome’s capacity to directly influence brain function (33). By integrating such insights with AI-driven analytics, the digital twin could identify microbial signatures predictive of neuropsychiatric conditions or sleep disturbances, enabling preemptive interventions (29, 141).

Technologically, this ecosystem would rely on neuralnanorobotics and multi-omics data fusion to achieve cellular-resolution monitoring. Neuralnanorobots, as proposed for brain-cloud interfaces, could theoretically navigate the gut-brain axis to detect microbial metabolites or synaptic activity, transmitting data via nanorobotic fiber optics to cloud-based supercomputers for real-time analysis (63, 142). Concurrently, wearable sensors and ingestible devices would capture circadian rhythms, gut motility, and immune markers, feeding into the digital twin’s adaptive algorithms (30, 143). The system’s predictive power would stem from mechanistic computational models, akin to those used in cardiovascular digital twins, which simulate individual physiology to uncover latent phenogroups and optimize therapeutic strategies (30, 144). Unsupervised machine learning could classify patients based on gut-microglia interactions, a critical node in neuroinflammatory disorders like Alzheimer’s disease, where microbiota modulate microglial activation states (28, 31).

The integration of DBS into the digital twin ecosystem is considered here as a long-term speculative application, despite the fact that it is a very invasive neurosurgical intervention that is outside the purview of nursing practice. DBS parameter change would continue to be solely under the supervision of neurologists or neurophysiologists in such future situations. Monitoring and coordination would be the main responsibilities of the nursing position, including tracking changes in sleep architecture, keeping track of patient reactions, and assisting with communication between the patient, the specialized team, and the digital twin system. By incorporating DBS device data into the patient’s unified health record, offering real-time visualization, and warning nurses of clinically significant deviations that need for expert assessment, the digital twin would facilitate this procedure.

The innovation of this ecosystem lies in its closed-loop adaptability. By coupling AI with real-time biosensing, the digital twin could dynamically adjust interventions—such as probiotic formulations, vagal nerve stimulation, or chronotherapy—to restore homeostasis. Vagal afferents, which detect intestinal nutrients and relay signals to the brain, offer a direct pathway for such modulation (63, 127). Experimental evidence shows that fat preference is mediated by distinct gut-brain circuits, suggesting that nutrient-specific pathways could be harnessed for metabolic interventions (63). Furthermore, the integration of explainable AI (XAI) ensures transparency in decision-making, addressing ethical concerns around algorithmic bias and clinical accountability (30, 129). Challenges, however, abound. The sheer complexity of gut-brain-sleep interactions demands federated data architectures to harmonize heterogeneous datasets while preserving privacy (78, 129). Regulatory frameworks must also evolve to address the dual-use potential of neuralnanorobotics and the ethical implications of enhancement versus therapy.

Long-term, this ecosystem could redefine precision medicine by bridging gaps between disparate disciplines. Transparent shadowing—a concept from brain-cloud interfaces—might be adapted to simulate patient-specific responses to dietary or pharmacologic interventions, fostering deeper clinician-patient collaboration (30, 142). The gut-microbiota-brain axis’s role in sleep regulation further underscores the potential to mitigate circadian disorders through microbial reprogramming (29, 141). Yet, speculative hurdles remain, including the scalability of neuralnanorobotics, the interpretability of multi-omic models, and the socio-economic disparities in accessing such advanced care (30, 78). Collaborative efforts among clinicians, data scientists, and ethicists will be pivotal to translating this vision into practice, ensuring that the gut-brain-sleep digital twin not only advances scientific understanding but also delivers equitable, patient-centric outcomes (29, 30).

While the AI-empowered gut-brain-sleep digital twin remains a forward-looking concept, its foundational pillars—neuralnanorobotics, microbiota modulation, and AI-driven phenomapping—are already under active investigation. By synthesizing these domains into a unified ecosystem, this approach could unlock unprecedented insights into the gut-brain axis, offering a paradigm shift in managing conditions from hepatic encephalopathy to insomnia. Future research must prioritize translational validation, ethical governance, and interdisciplinary convergence to realize its full potential (30, 63).

## 5 Digital-twin construction workflow

To construct the proposed CARE system and develop a cross-disciplinary research framework that engages both multi-specialty experts and key stakeholders, an additional working group—comprising professors with expertise in digital health and clinical specialists—must be tasked with forging collaborative networks with industry partners and potential investors. The development and implementation process of the CARE system leverages well-established methodologies from the architecture–engineering–construction–operation (AECO) industry—specifically, the integration of semantic information models with dense Internet of Things (IoT) sensor networks. These integrated tools work in tandem to achieve real-time synchronization between virtual twin replicas and the live physical states of target systems (145). By analogy, human-centric sensing devices—which generate high-resolution biological data, such as gut metagenomic sequencers, scalp electroencephalography (EEG) arrays, and comparable wearable or implantable monitoring tools—are integrated into a dedicated data processing layer (146). The multi-modal data streams generated by these devices facilitate the development of a dynamic, individual-specific digital twin—one whose state variables are updated at a millisecond-to-minute temporal cadence. This individual-specific digital twin, in turn, ensures the virtual model operates in lockstep with an individual’s real-time biological fluctuations. It also serves as a computable testbed for simulating and predicting biological outcomes across a range of therapeutic modalities—from pharmacologic interventions to lifestyle modifications.

AI technologies are now deeply integrated into the full digital twin lifecycle: specifically, reinforcement learning algorithms continuously calibrate model parameters against real-time streaming telemetry data, while convolutional neural networks (CNNs) and graph neural networks (GNNs) decode high-dimensional imaging datasets and omics-derived molecular signatures. The central engine for simulating cross-layer interactions in the suggested ecosystem is a HGNN-CF. This architecture facilitates interpretable causal inference through attention visualization and allows biologically faithful representation learning across heterogeneous node types. Within the proposed gut–brain–sleep digital twin ecosystem, these identical algorithmic frameworks ingest multimodal data meshes—including gut metagenomic readouts, neurophysiological time series, and sleep-stage hypnograms—to identify latent, non-linear coupling motifs between microbial metabolite flux, brain synaptic connectivity, and circadian rhythm dynamics. The inferred relational graph is subsequently embedded in a dedicated predictive analytics layer—one that flags pre-symptomatic deviations in biological trajectories. This layer further enables prospective risk stratification and delivers individualized, timing-sensitive intervention cues to the human host via closed-loop actuators integrated into the ecosystem (146).

**Figure 3:**
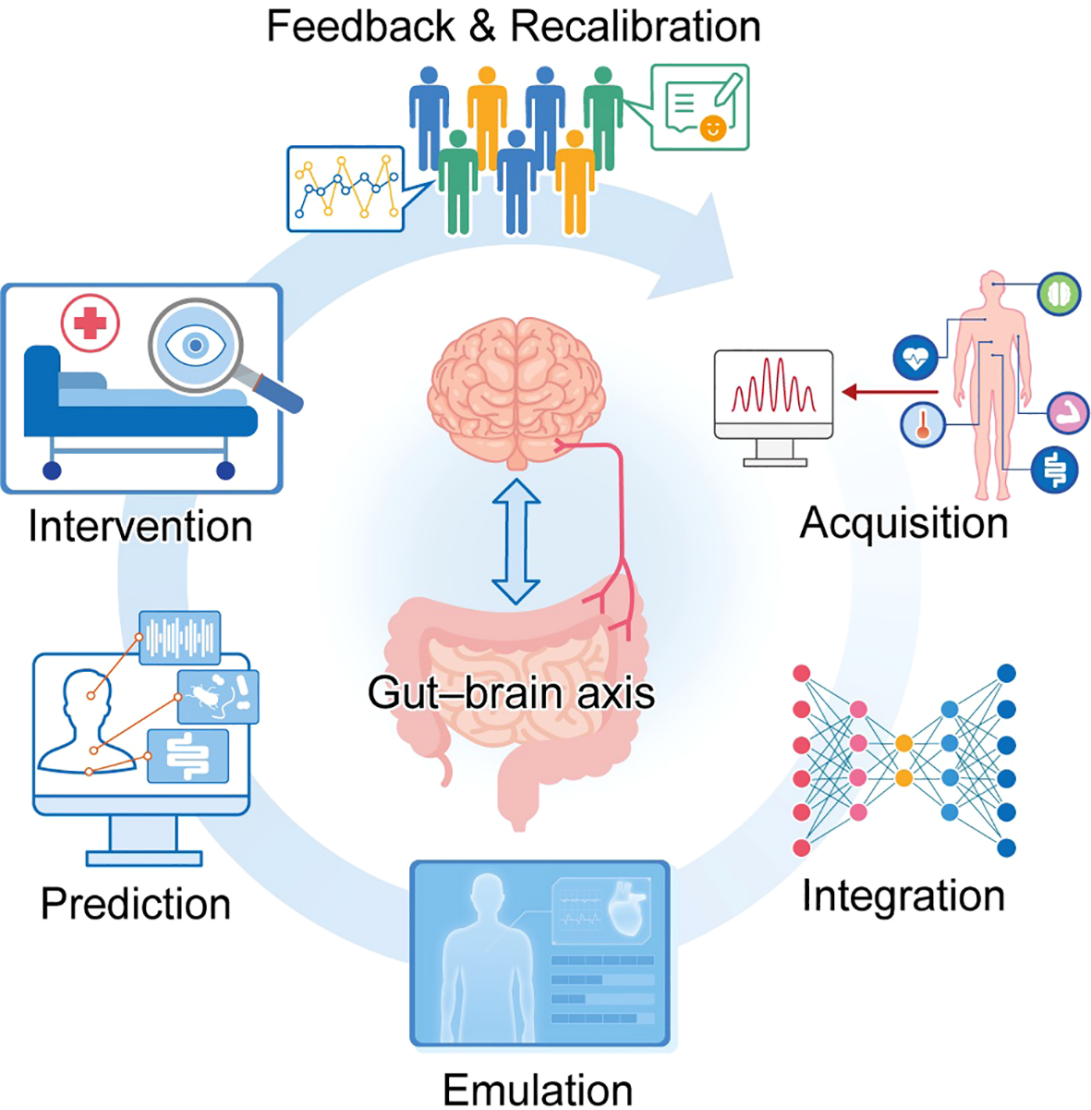
Operational workflow of the AI-enabled gut–brain–sleep digital-twin nursing ecosystem.

The data-driven construction workflow of the gut–brain–sleep digital twin ecosystem initiates with the acquisition of high-resolution, multi-source biological and physiological data—specifically, data streams from wearable health monitoring devices, continuous glucose monitoring (CGM) systems, and gut microbiome sequencing platforms. This workflow serves as a foundational component of the ecosystem, underpinning its ability to generate accurate virtual replicas (147) (**Figure 3**). Following processing, these data are incorporated into the DT framework, which uses sophisticated modeling techniques to forecast results and simulate physiological processes (148). The gut-brain axis and its role in neurological and sleep disorders, for example, can be better understood by using the DT to model how gut microbiota affects brain function and sleep quality (112). The AI component develops these models even further, through learning from longitudinal data, so the ecosystem can adjust, as patient condition changes, and gradually optimize interventions (149).

One of the main innovations of this ecosystem is its ability to deliver insights through real-time feedback loops. If the DT detects alterations in the gut microbiota composition linked to poor sleep, it can provide dietary recommendations or probiotic treatments to correct it (113). The system can suggest behavioral or medication changes to improve sleep quality, should it record abnormal brain activity while you sleep (114). In addition to improving patient outcomes, this dynamic approach empowers people to take an active part in managing their health (150).

Another important benefit of this ecosystem is its scalability. Compatibility and ease of adoption across various health care settings are ensured by the framework’s seamless integration into current EHR systems through the use of open-source platforms and interoperable health web standards. Additionally, the ecosystem’s modular design ensures its relevance and usefulness in the quickly changing field of digital health by enabling the addition of new data sources and functionalities as new technologies are developed (151).

## 6 Intervention modalities with the digital-twin ecosystem

It is especially promising to apply this ecosystem to nursing intervention modalities. This technology can help nurses, who are frequently at the forefront of patient care, provide individualized, evidence-based interventions. Nurses can take early action to avoid complications by using the ecosystem to notify them of subtle changes in a patient’s sleep or gut health. The digital twin can also be used as a teaching tool, enabling nurses to test out various care approaches in a mock setting prior to applying them in clinical settings. This lowers the possibility of unfavorable outcomes while simultaneously improving the quality of care (78, 152).

The ecosystem also responds to the increasing demand for affordable, scalable healthcare solutions. Larger patient populations can receive high-quality care thanks to the automation of data collection and analysis, which also lessens the workload for healthcare professionals. This is especially important when it comes to chronic illnesses, where ongoing care and monitoring are crucial. Continuous monitoring of the gut-brain-sleep interactions, may help patients with dementia or HE by enabling early disease progression detection and prompt treatment plan modifications (81, 153).

Research has focused on integrating nursing interventions to improve sleep in critical and intensive care settings, with a particular emphasis on therapeutic modalities and environmental changes. In neurocritical care units, Olson et al. showed that staff members’ focused efforts to reduce outside distractions during designated quiet periods greatly increase the likelihood that patients will fall asleep (154). Similarly, according to Richardson et al.’s evaluation of the use of eye masks and earplugs, these easy ways to reduce exposure to light and noise can potentially improve the quality of sleep for patients with high dependency (155).

Building on the digital twin’s predictive capabilities, the ecosystem supports a comprehensive suite of non-invasive interventions that fall firmly within nursing’s scope of practice. In order to suggest the best times for bright light exposure and light limitation, AI algorithms examine patient sleep-wake patterns, chronotype, and wearable-derived activity data. Based on these suggestions, IoT-enabled lighting systems automatically modify color temperature and intensity. Nurses set up procedures, use the digital twin dashboard to track adherence, and modify schedules in response to patient feedback. The ecosystem incorporates evidence-based digital CBT-I modules, such as cognitive restructuring, stimuli management, and sleep restriction. AI uses wearable data and patient-reported outcomes to tailor module distribution. While the digital twin monitors progress and links adherence with changes in sleep architecture, nurses receive signals for patients who need escalation and offer focused coaching.

AI models use individual circadian rhythms, sleep patterns, and gut microbiome dynamics to predict the best times to administer prebiotics, probiotics, and postbiotics. The digital twin tracks adherence, provides dosing reminders, and integrates metabolite data from non-invasive VOC sensors to evaluate efficacy while nurses oversee supplementation protocols. tVNS is a non-invasive neuromodulation technique that modifies inflammatory tone and sleep architecture by stimulating the vagus nerve’s auricular branch. The intervention can be used in accordance with nursing protocols, and the digital twin’s real-time efficacy feedback can be used to determine the length and timing of sessions. Environmental changes and sensory interventions that nurses can use at the patient’s bedside are supported by the ecosystem. Relaxation techniques are delivered through the digital twin’s patient interface, with nurses providing guidance and monitoring outcomes.

Aromatherapy and other complementary therapies have also been investigated for their potential to improve anxiety and encourage sleep. Aromatherapy may be used as a stand-alone nursing intervention to help ICU patients having percutaneous coronary interventions sleep better and feel less anxious (156). Neurophysiological methods have been studied in addition to environmental and sensory interventions. Welch et al. discovered that family nurture interventions, which promote peaceful and affective communication, raised the electroencephalographic activity linked to sleep in preterm infants, pointing to a possible way to improve sleep through relational and emotional support (157).

The potential of cutting-edge technological interventions to alter sleep quality has drawn attention, especially those that make use of neurofeedback and brain training. Self-regulation of brain activity may be a promising modality for improving sleep in vulnerable populations, as evidenced by Laborda-Sánchez et al.’s demonstration that neurofeedback training could improve sleep among elderly women in nursing homes (158). Effendy et al. explored brain gym exercises, finding improvements in sleep quality and reductions in anxiety among elderly individuals, further supporting the utility of neuro-motor interventions (159).

Emerging research underscores the significance of the gut–brain axis in sleep regulation, with the gut microbiota playing a pivotal role. Evidence indicates that gut microbiota and sleep engage in bidirectional molecular dialogue, and that targeted microbial modulation—whether via precision probiotics, synbiotic blends or fecal metabolite mimetics—can restore sleep continuity, deepen slow-wave activity and normalize circadian timing, thereby ameliorating sleep disturbances (7). This is corroborated by studies examining microbiome signatures and their influence on mental health and sleep. Pedroso et al. identified associations between gut microbiome profiles, genetic factors, and improvements in sleep and mental health symptoms following personalized digital care, emphasizing the microbiome’s therapeutic potential (160).

A new avenue for tailored nursing interventions is provided by the incorporation of digital therapies based on genomic and microbiome data. A future where AI-powered digital ecosystems could customize interventions based on individual microbiota and genetic profiles is suggested by Pedroso et al.’s demonstration that microbiome and genetic markers could predict and improve mental health and sleep outcomes (160).

The benefits of massage therapy for sleep and associated symptoms have been studied in addition to microbiome-focused approaches. In patients with multiple sclerosis, Gaballah et al. discovered that effleurage massage could lessen anxiety, pain, fatigue, and sleep disturbances. This suggests that manual therapies are still useful in holistic nursing care (161).

When taken as a whole, these studies show a variety of strategies for enhancing sleep, including sensory interventions, environmental changes, neurophysiological methods, and treatments that target the microbiota. A promising path for individualized, comprehensive nursing interventions is the growing role of AI-powered digital twin ecosystems integrating gut-brain-sleep interactions, which may improve overall health outcomes and sleep quality in a variety of patient populations.

## 7 Real-world validation landscape

The integration of digital twin technology with the biological and behavioral health domains is the foundation of the developing field of AI-powered “gut–brain–sleep” digital-twin nursing ecosystems. By highlighting a “digital-twin as-a-service” approach that can encompass multiple strategic assets within health organizations, Ricci et al. draw attention to the conceptual shift from isolated digital assets to interconnected ecosystems of digital twins (162). This fundamental viewpoint facilitates the creation of thorough, practical validation frameworks for intricate health ecosystems, such as those pertaining to the gut-brain-sleep axis.

Studies on the gut-brain-microbiota axis highlight how important it is for controlling sleep and maintaining general health. Sen et al. investigate the molecular processes through which microbiota affect sleep, highlighting the significance of comprehending interactions within the microbiome (6). Experimental data further delineate a reciprocal sleep–microbiota axis: controlled manipulation of microbial composition and metabolic output—through calibrated prebiotic substrates, live biotherapeutic consortia, or postbiotic metabolites—systematically enhances slow-wave stability, increases spindle density, and consolidates circadian sleep architecture, thereby conferring measurable therapeutic gains in sleep quality (7). A clinical study maps dysbiotic gut signatures—contracted α-diversity, depleted butyrate producers, and pro-inflammatory blooms—to polysomnographic deficits in insomnia and sleep apnea, then embeds these microbial phenotypes within a digital-twin engine that uses AI-driven optimization to design individualized prebiotic, probiotic, or postbiotic regimens aimed at restoring microbial eubiosis and normalizing sleep architecture (8).

From infancy through old age, the brain-gut-immune axis affects sleep-wake cycles, according to Sgro et al.’s developmental and lifespan perspective (163). By simulating the intricate relationships across various life stages, digital twins can help with dynamic, real-time monitoring and validation in diverse populations. These models can simulate health trajectories and forecast the results of interventions by incorporating immune, microbiota, and neural data.

Important biochemical insights into gut-brain communication pathways are provided by metabolomics studies (164). The biochemical landscape provided by their high-coverage metabolomics profiling of germ-free versus conventional mice can guide the creation of AI models in digital twins, allowing for accurate simulation of the effects of microbes on host metabolism and brain function.

Developments in simulation and visualization technologies are also advantageous to the validation landscape. Kikuchi et al. show how augmented reality combined with city digital twins can improve stakeholder participation and decision-making (165). Similar visualization techniques may make it possible for patients and clinicians to engage with digital twins of the gut-brain-sleep system in the healthcare setting, allowing for personalized intervention planning and real-world validation.

The convergence paradigm of AI and digital-twin architectures is illustrated by two strands: one deploys virtualized radio environments to emulate large-scale wireless traffic in real time, the other couples high-resolution dietary logs to longitudinal metagenomic trajectories, demonstrating how in-silico replicas can both optimize network performance and predict microbiota-mediated health outcomes (166). These studies demonstrate how interventions within digital twin ecosystems could be validated in real-time by AI, guaranteeing that models appropriately capture biological and environmental variability.

Through interdisciplinary insights into metabolic pathways, developmental dynamics, visualization technologies, and microbiota interactions, the validation landscape for AI-powered gut-brain-sleep digital-twin nursing ecosystems is progressing. Together, these advancements facilitate the development of solid, empirically validated models that can be used in integrated digital twin ecosystems for therapeutic intervention and personalized health management.

This ecosystem’s capacity to anticipate and reduce risks related to dysregulation of the gut, brain, and sleep is one of its main technological advantages. Microbial abundance data can be analyzed by AI algorithms to predict dysbiosis trajectories, which could result in sleep disorders or other negative neurodevelopmental outcomes (31, 167). Models of generative AI like Q-nets that replicate ecosystem dynamics and identify patterns absent by earlier models have increased this intelligence (168). The universe of real-world validation of ecosystem predictions reduces the risks of bias that stem from clinical or laboratory settings. Benefits are backed up by actual data derived from heterogeneous patient populations in natural settings (28, 29). The system also solves the privacy issue of the patient through strong data encryption and access control to ensure secure handling of private health data while allowing ongoing monitoring (33).

This ecosystem is used for many applications, especially in chronic disease management and precision medicine. By imitating the effects of dietary changes, probiotics, or drug interventions at the gut-brain-sleep axis, it can permit the design of personalized treatments for patients with disorders of gut-brain interaction (DGBIs), like HE or IBS (63, 169). The ecosystem can be applied to a variety of medical imaging modalities, illnesses, and organs because of its modular design, which enables it to generalize beyond particular circumstances (141). In light of the demographic shift toward an aging population, where long-term monitoring and early disease prediction are critical to improving patient outcomes, this flexibility is especially crucial (152).

## 8 Ethical, legal & social implications

The AI-powered digital twin ecosystem presents ethical issues despite its potential, especially with regard to consent and data privacy. Strong security measures are required to preserve patient confidentiality when gathering and analyzing extremely sensitive health data, such as genetic information, microbiome profiles, and neural activity (48, 79). The necessity for open data governance frameworks is highlighted by the possibility that discrimination, stigmatization, or misuse of personal information could result from data breaches or illegal access (162, 163). Informed consent procedures need to be modified to guarantee that patients are fully aware of the ramifications of using such a system, including the possibility of algorithmic biases or unforeseen consequences (48, 164). The implementation of this ecosystem is also heavily influenced by legal issues. AI-driven health technologies present special challenges that current regulatory frameworks are ill-prepared to handle, especially when it comes to digital twins. Unresolved issues about liability in situations involving inaccurate forecasts or unfavorable results call for the creation of new legal guidelines that strike a balance between creativity and responsibility (79, 163). The digitization of healthcare services remains a contentious issue—particularly when vulnerable or underserved populations lack equitable access to the necessary digital infrastructure, tools, or literacy support. Policymakers, in turn, must proactively develop strategies to mitigate delays in the deployment of digital health technologies and address barriers to adoption among socioeconomically disadvantaged communities (163).

The digitization of healthcare services, while offering efficiency gains, remains contentious—particularly when underserved groups lack equitable access to essential digital resources. To address this, policymakers must iteratively design strategies to reduce delays in digital health technology rollouts and mitigate adoption barriers for socioeconomically marginalized communities (28, 163). The integration of AI-driven predictive tools into clinical practice risks shifting clinicians’ roles from primary decision-makers to facilitators of AI outputs—a trend that raises critical questions about the potential erosion of human clinical judgment in healthcare delivery (79). To mitigate these risks technologists, ethicists and clinicians need to be encouraged to work together across disciplines, to build an ecosystem which can complement humans rather than substitute them (163).

Additionally, incorporating digital twins into nursing procedures has the potential to enhance patient care. This ecosystem has the potential to improve clinical decision-making, optimize workflows, and lower the risk of errors by giving nurses access to real-time, data-driven insights (48). By remotely monitoring patients’ sleep habits and digestive health with digital twins, nurses can take proactive measures to avoid complications (79, 162). However, implementing such technologies necessitates a cultural shift toward data-driven care models and a large investment in infrastructure and training (48).

The need to align technological advancements with established human rights and professional standards is highlighted by the legal and ethical foundations guiding health care innovations, especially in genomics (165). When taking into account AI-driven systems that track and modify private biological and behavioral data in a “gut–brain–sleep” framework, this alignment becomes even more relevant. Since these systems may affect people’s privacy and autonomy, strict ethical supervision is required.

The infrastructure requirements for ethically sensitive scientific discoveries are best illustrated by social mining and big data ecosystems. In order to manage the social ramifications of AI in nursing ecosystems, responsible data sharing and analysis are made easier by the creation of open, integrated platforms. The social acceptance of AI-enabled health interventions depends on addressing societal concerns about data security, consent, and equitable access, all of which these platforms can assist with. Particular moral dilemmas surrounding brain death in pregnancy emphasize how crucial it is to set precise rules for handling difficult situations involving decisions about life support (166). The incorporation of AI into the “gut-brain-sleep” ecosystem also needs to take ethical limits on decision-making autonomy into account, particularly when AI systems have a significant impact on important health outcomes. To preserve public confidence in such delicate situations, ethical compliance is essential. Research on sleep interventions, like brain gym exercises, highlights how crucial ethical approval and following set protocols are in clinical studies (151). The need for ethical rigor in the development and application of these technologies is highlighted by applying comparable standards to AI-based interventions in the gut-brain-sleep axis, particularly with regard to patient safety and informed consent. Examined in the context of accounting, the impact of emerging technologies on moral decision-making demonstrates how technological advancements can both reduce and increase moral risks (167). AI’s ability to process large amounts of data and make decisions automatically could help the healthcare industry by reducing human error, but if left unchecked, it could also lead to ethical blindness. This emphasizes how crucial it is to incorporate ethical issues into the governance and design of AI systems.

A thorough analysis of AI applications in nursing care demonstrates the growing interest in incorporating AI while recognizing the related social, legal, and ethical concerns (168). These concerns, which are especially important in systems that track and affect behavioral and biological health metrics within a “gut–brain–sleep” ecosystem, include data privacy, accountability, and the possibility of bias.

The gut microbiota and sleep exhibit a bidirectional, functional interaction—meaning perturbations to one system can influence the other. Consequently, targeted modulation of the gut microbiota holds potential for improving sleep quality in populations with sleep disturbances (7). The integration of artificial intelligence (AI) technologies into gut microbiota-sleep research and clinical practice offers significant potential for advancing personalized therapeutic strategies. The AI-driven integration also raises critical ethical considerations—specifically, challenges related to informed patient consent, the secure and ethical stewardship of sensitive microbiome data, and the responsible oversight of microbiota modulation interventions. Recent advancements in AI applications within neuro-oncology present both opportunities and challenges for the broader field of gut microbiota-sleep research—serving as a paradigmatic example of how technological progress in brain health research can inform adjacent domains (169). These observations hold relevance for the gut-brain-sleep digital twin ecosystem—specifically, they underscore the need to balance three critical priorities: ethical stewardship of sensitive patient data, robust informed consent protocols, and maintenance of therapeutic efficacy—when integrating AI tools for the diagnosis and treatment of neurological and gastrointestinal (GI) disorders. A systematic review examining AI applications in neuro-oncology emphasizes the critical role of interdisciplinary research—bringing together clinicians, data scientists, ethicists, and patient advocates—in addressing both the ethical dilemmas and clinical translational challenges posed by this technology (170). From the perspective of the proposed “gut-brain-sleep” digital twin ecosystem, this perspective argues for the need for a systematic, interprofessional framework to address ethical, legal, and social issues (ELSS) when implementing AI-driven digital twins within nursing practice. Specifically, when integrating AI components into this “gut-brain-sleep” integrated digital-twin nursing ecosystem, three core domains require deliberate consideration: foundational ethical principles, established legal frameworks, and broader social impacts. To facilitate the responsible development and deployment of these AI-integrated digital twin systems, three key practices are essential: strict adherence to standardized operational protocols, transparent governance mechanisms, and regular interdisciplinary dialogue. These practices collectively serve to safeguard human rights and ensure equitable access to high-quality, AI-enhanced healthcare.

## 9 Future prospects

The incorporation of AI and quantum computing into a “gut-brain-sleep” digital-twin nursing ecosystem is an innovative development in personalized healthcare. The novel framework facilitates unprecedented insights into the gut-brain axis and health and disease consequences by modelling the microbiome-behavior-sleep entanglement, which has a million dimensions, under quantum simulations. By developing a digital twin of someone’s microbiome and brain activity, this ecosystem enables real-time monitoring of health outcomes, predictive analytics, and personalized interventions (11, 170).

A bidirectional communication network that connects the central nervous system and the gastrointestinal system, the gut-brain axis is essential for controlling behavior, thought, and sleep. Numerous neurodevelopmental and psychiatric conditions, such as schizophrenia, autism spectrum disorder, and migraines, have been linked to dysbiosis, or microbial imbalance (14, 18). The intricacy of microbial ecosystems and the computational difficulties of evaluating high-dimensional data have restricted the use of traditional methods to investigate these interactions. However, AI-driven models, like the Q-net framework, have shown a high degree of accuracy in predicting the dynamics of microbial abundance and identifying risk factors for suboptimal neurodevelopment (11).

The suggested digital-twin ecosystem combines a number of cutting-edge technologies, such as wearable sensors, and cloud-based supercomputing, to produce a dynamic and thorough model of a person’s health. Continuous data on physiological and behavioral metrics is provided by wearable sensors, and cloud-based platforms allow for the safe and scalable processing, analysis, and storage of this data (13, 171). These technologies work together to create a closed-loop system that not only forecasts health outcomes but also suggests and carries out individualized interventions, like dietary changes, probiotic therapies, or neuromodulation techniques, to improve sleep and cognitive function and restore microbial balance (11, 14).

An observation on gut dysbiosis-associated systemic inflammation and cognitive decline may yield an early biomarker for neurodegenerative diseases, such as Alzheimer’s (172). It may predict the efficacy of treatments that target the microbiota (e.g., fecal microbiota transplantation, synbiotics) in alleviating symptoms of mental disorders or improving sleep quality (17).

The quantum computing-powered AI “Gut-Brain-Sleep” Digital Twin nursing ecosystem embodies a paradigmatic shift in modern healthcare delivery—one that redefines how personalized, data-driven care is designed and implemented for sleep-microbiome-related health conditions. By integrating advanced technologies—including quantum computing frameworks, multi-omics analytical models, and real-time sensor data integration—the ecosystem establishes a personalized analytical mechanism. This mechanism, in turn, deciphers the intricate, bidirectional relationships between modifiable health behaviors, sleep architecture, and gut microbiome composition. By addressing a spectrum of objectives—from early disease detection and prevention to the optimization of targeted therapeutic strategies—this ecosystem renders feasible a future of truly predictive, preventive, and personalized healthcare. Notably, the gut-brain-sleep axis emerges as a particularly promising therapeutic target—given the growing body of empirical evidence that underscores its regulatory role in circadian rhythm homeostasis, cognitive function, and emotional well-being (173). The gut microbiota exerts a regulatory influence on sleep quality through the biosynthesis of neuroactive metabolites and the modulation of neurotransmitter levels, including serotonin and gamma-aminobutyric acid (GABA) (173). Conversely, poor sleep quality can induce perturbations in gut microbial composition and diversity—perpetuating a bidirectional vicious cycle. This cycle contributes to the development of neuropsychiatric symptoms and gastrointestinal dysfunction, which in turn further exacerbate sleep disturbances (173). The proposed “gut-brain-sleep” digital twin model is uniquely designed to capture the dynamic, bidirectional interactions between sleep disorders and their downstream cascading effects on brain health—including impacts on neuroinflammation, synaptic plasticity, and circadian rhythm regulation. Building on this, clinicians can harness the insights generated by this digital twin model to design and implement tailored, evidence-based interventions—such as personalized sleep hygiene protocols, microbiota-targeted dietary adjustments, or timed pharmacologic interventions—for patients with sleep-brain-microbiome axis dysregulation.

A key innovation of this “gut-brain-sleep” digital twin nursing ecosystem lies in its ability to enable nursing professionals to design patient-specific, evidence-based treatment plans. Specifically, the model is capable of simulating how targeted perturbations—including dietary modifications, probiotic supplementation regimens, or pharmaceutical interventions—impact gut microbial composition and diversity, which in turn modulate sleep architecture and cognitive function (28). This simulation capability is particularly valuable for managing complex, chronic conditions—such as fibromyalgia and IBS—given that the gut-brain axis plays a pivotal role in regulating the symptoms of these disorders (82, 153). Additionally, the system’s capacity to track and monitor patient responses to interventions in real time facilitates the continuous assessment of treatment efficacy. This, in turn, enables timely adjustments to therapeutic strategies—ultimately improving overall clinical outcomes (31, 87).

The future roadmap for this “gut-brain-sleep” digital twin nursing ecosystem envisages the implementation of a dedicated randomized controlled trial (RCT)—dubbed the “Digital Twin Nursing Intervention (D-TWIN-RCT)”—to empirically validate the ecosystem’s clinical effectiveness and real-world scalability. A rigorous methodological design framework will be employed to assess the impact of digital twin-guided interventions on key outcome measures—including patient-reported quality of life (QoL), objective cognitive function metrics, and sleep quality assessments (31, 87). With standard-of-care (SoC) interventions serving as the control arm, the D-TWIN-RCT seeks to demonstrate that the digital twin-enabled nursing approach offers superior accuracy in predicting treatment responses, enhanced clinical effectiveness, and greater patient acceptability compared to conventional care. Additionally, the D-TWIN-RCT will evaluate the integration of wearable sensing devices and Internet of Things (IoT) technologies—with two primary goals: first, to enhance real-time data collection efficiency and remote patient monitoring capabilities; second, to expand the ecosystem’s telemedicine capacity for delivering decentralized care (31, 43). To guarantee the safe handling of private patient data, strong encryption procedures, anonymization strategies, and adherence to GDPR and ISO 27001 standards will be necessary (31, 87). The D-TWIN-RCT’s inclusion of a variety of patient populations will lessen biases and guarantee that the results are broadly applicable (31, 44).

## 10 Conclusion

By combining the dynamical ecology of the gut microbiome with the neuro-immune regulation of sleep and cognition, we suggest that the next frontier in precision nursing is not the incremental digitization of current tasks but rather the creation of a living, self-evolving digital twin. This vision is made possible by the conceptual, computational, and clinical scaffolding provided by the G-B-S DT-N ecosystem that is being presented here. Through the integration of strain-level microbiome chronobiology, EEG micro-architecture, and momentary affect into an explainable AI framework that quantifies uncertainty, nurses are able to predict, in hours to days, when dysbiosis, sleep fragmentation, or affective relapse will occur. Sensitive information is kept in patient-specific enclaves while federated edge learning guarantees that this insight is applicable to other populations. Iteratively distilling experiential “nursing intuition” into algorithmic memory—a knowledge asset that neither doctors nor engineers can duplicate—the nurse-in-the-loop reinforcement paradigm transforms each bedside acceptance or rejection of an AI recommendation into labelled policy gradients. The ecosystem might shift the focus of nursing from providing reactive care to actively designing the ecosystem, making microbiome-precision sleep health a new area of advanced practice nursing. The G-B-S DT-N paradigm, in the end, transforms the theoretical microbiota-gut-brain axis into an operational, morally guided, nurse-led engine for precision health in the twenty-first century.
