# Supplementary material for: AI digital-twin ecosystem translating gut-microbiome–neuroimmune signals into precision sleep–mood interventions
Source: Front Psychiatry. 2026 Apr 15;17:1703605. doi: 10.3389/fpsyt.2026.1703605 (PMC13125875; doi:10.3389/fpsyt.2026.1703605)
Supplement: Supplementary file 1 [file Table1.docx]

Microbial Deficit Risk (Mδ) - Definition and Calculation

The microbial deficit risk (Mδ) is a composite metric that quantifies the functional integrity of the gut microbiome. It is calculated as a weighted sum of four complementary dimensions:


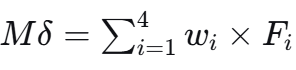


| Component | Definition | Weight (wᵢ) |
| --- | --- | --- |
| **F₁: Alpha Diversity** | Inverse Simpson index calculated from 16S rRNA sequencing data; reflects microbial richness and evenness. | 0.25 |
| **F₂: Functional Balance Ratio** | Log-transformed ratio of butyrate-producer abundance (Faecalibacterium prausnitzii + Roseburia spp.) to pro-inflammatory Enterobacteriaceae abundance. Higher values indicate healthier gut ecology. | 0.30 |
| **F₃: Butyrate Production Capacity** | Predicted butyrate synthesis rate (μmol/g feces/day) derived from metagenomic pathway analysis (e.g., butyrate kinase, butyryl-CoA transferase pathways). | 0.30 |
| **F₄: Bacterial Diversity Loss Index (BDI)** | Composite measure of keystone species extinction risk, calculated from the disappearance of core taxa relative to baseline. | 0.15 |
| Interpretation Thresholds:  Mδ < 0.3: Normal microbial function – no intervention required.  0.3 ≤ Mδ ≤ 0.6: Moderate risk – yellow-tier alert; lifestyle or dietary modifications recommended.  Mδ > 0.6: Severe dysbiosis – red-tier alert; consider fecal microbiota transplantation (FMT) or intensive probiotic intervention. | | |

The weighting scheme is implemented within the Q-net generative AI framework, which continuously learns optimal weights from incoming clinical data to dynamically adapt the metric to specific patient populations and clinical contexts (e.g., higher weight for F₃ in sleep disorder patients). All components are normalized to age-matched reference populations to account for age-related microbial variation.
